# The tumor ecosystem in head and neck squamous cell carcinoma and advances in ecotherapy

**DOI:** 10.1186/s12943-023-01769-z

**Published:** 2023-04-06

**Authors:** Yingying Gong, Lisha Bao, Tong Xu, Xiaofen Yi, Jinming Chen, Shanshan Wang, Zongfu Pan, Ping Huang, Minghua Ge

**Affiliations:** 1 Center for Clinical Pharmacy, Cancer Center, Department of Pharmacy, Zhejiang Provincial People’s Hospital (Affiliated People’s Hospital, Hangzhou Medical College), Hangzhou, China; 2 Otolaryngology & Head and Neck Center, Cancer Center, Department of Head and Neck Surgery, Zhejiang Provincial People’s Hospital (Affiliated People’s Hospital, Hangzhou Medical College), Hangzhou, China; 3 Key Laboratory of Endocrine Gland Diseases of Zhejiang Province, Zhejiang Provincial People’s Hospital, Hangzhou, China; 4 Clinical Research Center for Cancer of Zhejiang Province, Hangzhou, People’s Republic of China

**Keywords:** Head and neck squamous cell carcinoma, Tumor ecosystem, Ecological therapy

## Abstract

The development of head and neck squamous cell carcinoma (HNSCC) is a multi-step process, and its survival depends on a complex tumor ecosystem, which not only promotes tumor growth but also helps to protect tumor cells from immune surveillance. With the advances of existing technologies and emerging models for ecosystem research, the evidence for cell-cell interplay is increasing. Herein, we discuss the recent advances in understanding the interaction between tumor cells, the major components of the HNSCC tumor ecosystem, and summarize the mechanisms of how biological and abiotic factors affect the tumor ecosystem. In addition, we review the emerging ecological treatment strategy for HNSCC based on existing studies.

## Introduction

Head and neck squamous cell carcinoma (HNSCC) is a heterogeneous disease and includes squamous cell carcinomas derived from pharynx. Its incidence rate ranks 7th among common cancers worldwide. The incidence of HNSCC continues to rise and is anticipated to increase by 30% by 2030 [1]. More than 50% of HNSCC patients suffered from recurrence and metastasis within three years [1, 2]. Lung is the most frequent site of distant metastasis in HNSCC, accounting for 70–85% of the cases [3]. Some major pathogenic factors of HNSCC have been recognized in the past several decades. The occurrence of HNSCC is commonly related to prior infection with oncogenic virus of human papillomavirus (HPV) which is a part of the tumor ecosystem [4]. Although surgery, radiation therapy (RT), and chemotherapy are the main treatment strategies for HNSCC patients, the 5-year survival rate for HNSCC has barely improved over the past 30 years [5]. Few options available to treat recurrent or metastatic (R/M) HNSCC also contributes to its poor prognosis [2].

The high malignancy and poor prognosis of HNSCC are closely related to the complexity of its ecosystem. Cancer is increasingly recognized as a “tumor ecosystem,” in which tumor cells work with other tumor cells and host cells in their microenvironment to adapt to varying conditions [6, 7]. From a holistic perspective, the tumor ecosystem can be viewed as an intersection of the host-tumor ecosphere. In this ecosphere, “living organisms” and their local/distal “living habitats” together with interior/exterior stimuli (Table 1) jointly promote the aggressiveness and progression of cancers [8, 9]. In HNSCC, tumor cells cooperate with living organisms/abiotic factors to facilitate progression of cancer cells. Therefore, deciphering the complexity of HNSCC and its ecosystem maybe the foundation for establishing early diagnosis and creating effective and precise treatments.

**Table 1:** Various cytokines in the tumor ecosystem of HNSCC

Stimulating factors	Secreting cells	Biological functions	Activating mechanisms	Refs
**Chemokine**				
CCL2	Tumor cell	Drive macrophages to M2-type transformation	**-**	[51]
CCL13	TAM	Promote tumor metastasis	**-**	[53]
CCL18	TAM	Promote tumor metastasis and invasion	**-**	[54]
CXCL1	CAF	Promote tumor metastasis	**-**	[175]
CXCL8	Tumor cell	Improve the survival rate and angiogenic potential of endothelial cells	AKT signaling pathway	[95]
CXCL12	CAF	Recruit TAMs, drive macrophages to M2-type transformation	**-**	[176]
CXCL13	Follicular dendritic cell	Recruit B cells	**-**	[177]
**Chemokine receptor**				
CCR2	Tumor cell	Drive macrophages to M2-type transformation	**-**	[51]
CXCR2	Monocyte	Recruit neutrophils	**-**	[88]
**Growth factor**				
BDNF	CAF	Promote tumor metastasis	TrkB signaling	[178]
EGF	Tumor cell	Improve the survival rate and angiogenic potential of endothelial cells	ERK signaling pathway	[95]
EREG	CAF	Promote tumor invasion	JAK2-STAT3 signaling pathway	[179]
HGF	CAF	Promote tumor metastasis	HGF/c-Met signaling pathway	[180]
TGF-β	CAF	Induce EMT	-	[24, 27]
		Drive macrophages to M2-type transformation	-	[45]
		Promote tumor metastasis	SOX9	[181]
	TAM	Promote tumor invasion	-	[55]
		Mediate immunosuppression	-	[57]
	B cell	Anti-tumor immunity	-	[77]
	MDSC	Promote PMN formation and metastasis	-	[83]
	Tumor cell	Promote inflammation, angiogenesis, and epithelial hyperproliferation	-	[97]
VEGF	TAM	Stimulate angiogenesis	-	[51]
	MDSC	Promote PMN formation and metastasis	-	[83]
	Tumor cell	Enhance the migration of tumor cells and protect them from apoptosis	AKT/ERK signaling pathway	[182]
		Mediate M2 TAMs	-	[98]
**Inflammatory cytokine**				
IL-1β	CAF	Promote tumor invasion	-	[175]
	Tumor cell	Promote activation between CAFs and tumor cells	CXCL1	
	Immune cell	Increase Th17 cells	-	[67]
IL-6	Endothelial cell	Enhance the survival, self-renewal and tumorigenic potential of CSCs	STAT3 signaling pathway	[25]
		promote tumorigenesis	JAK2/STAT3 signaling pathway	[44]
	TAM	Drive macrophages to M2-type transformation	-	[52]
	Tumor cell	Increase plasma NETs	-	[92]
		Improve the survival rate and angiogenic potential of endothelial cells	STAT3 signaling pathway	[95]
IL-10	CAF	Drive macrophages to M2-type transformation	-	[45]
	TAM		-	[51]
		Mediate immunosuppression	-	[57, 59]
	B cell	Anti-tumor immunity	-	[77]
IL-23	Tumor cell and TIL	Increase Th17 cells	-	[67]
IL-35	B cell	Anti-tumor immunity		[77]
MIF	Tumor cell	Recruit neutrophils	-	[88]
TNF-α	Macrophage	Increase plasma NETs	-	[92]

CAF, cancer-associated fibroblast; CSC, cancer stem cell; MDSC, myeloid-derived suppressor cell; NET, neutrophil extracellular trap; PMN, pre-metastatic niche; TAM, tumor-associated macrophage; Th17, T helper 17; TIL, Tumor infiltrating lymphocyte

Herein, we focus on recent advances in the main components of the HNSCC tumor ecosystem and the crosstalk between them. Through a detailed interpretation of the tumor ecosystem, we summarized the ecologically rational strategies for HNSCC treatment and their possible deficiencies.

## The components of the ecosystem in HNSCC

In view of the heterogeneity of cell phenotypes and cellular relationships in HNSCC, the entire tumor ecosystem should be taken into consideration when classify and treat patients. However, different cell types which express certain genes cannot be identified by traditional RNA sequencing (RNA-seq) which can only provide a virtual average of the various cellular components [10]. Single-cell RNA-seq (scRNA-seq), a valid method developed in recent years, which allows researchers to reveal the function and state of individual cell, enables the dissection of heterogeneous tumors and elucidation of the components within the tumor ecosystem [11–14]. Individual tumors are characterized by cellular diversity, i.e., as malignant or stromal cells [15]. Based on scRNA-seq, researchers discovered that non-malignant cells in HNSCC were grouped into eight main clusters: cancer-associated fibroblasts (CAFs), tumor-associated macrophages (TAMs), T cells, B/plasma cells, mast cells, dendritic cells, endothelial cells, and myocytes [16].

**Fig. 1:**
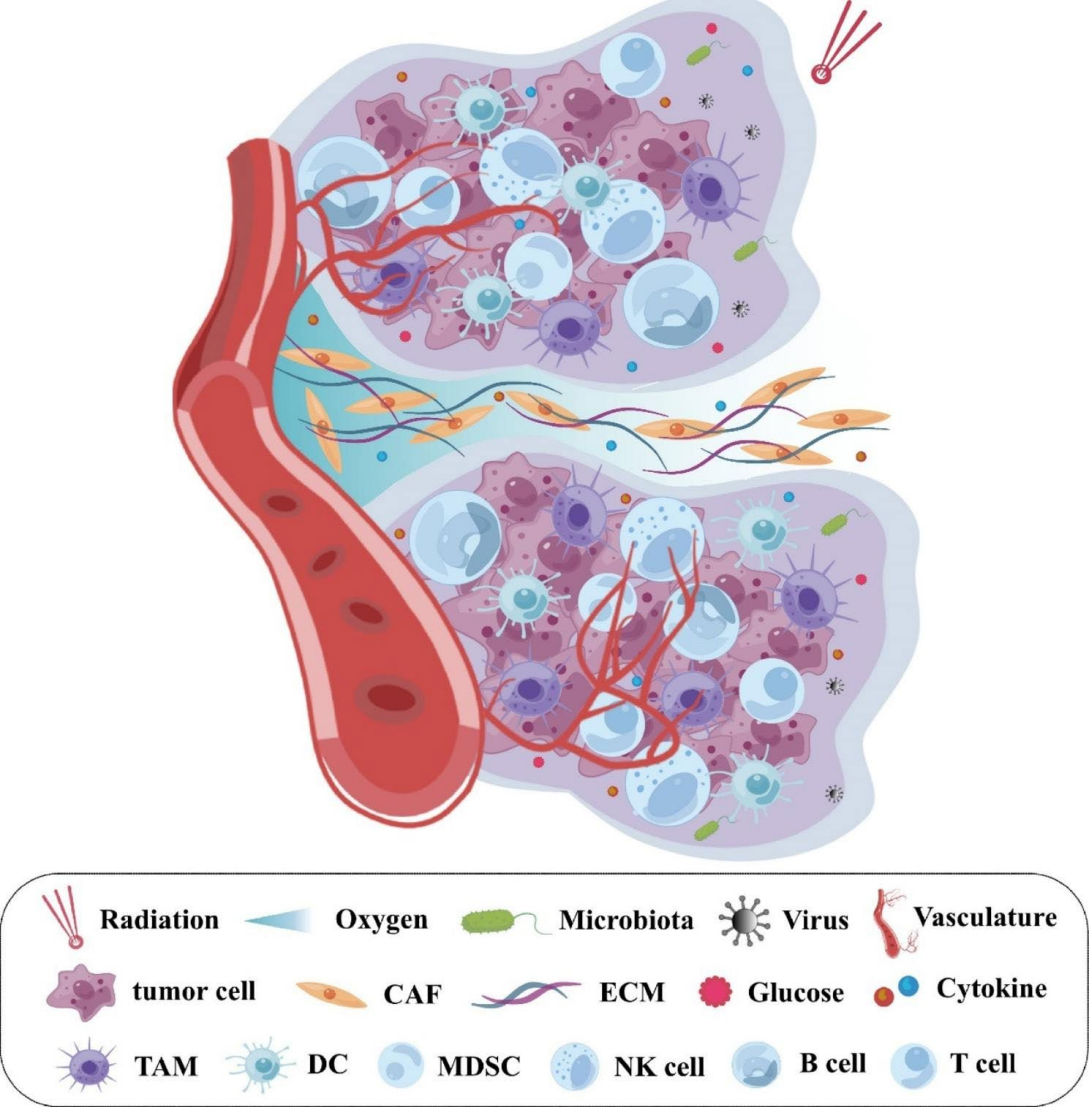
Tumor ecosystem in HNSCC. The tumor is a complex ecosystem composed of various cell types and microorganisms (e.g., HPV and bacterium). Besides biological factors, ECM, energy, oxygen, and therapeutic interventions (e.g., radiation) serve as abiotic factors that also shape the tumor ecosystem. Abbreviations: CAF, cancer-associated fibroblast; DC, dendritic cell; ECM, extracellular matrix; HPV, human papillomavirus; HNSCC, head and neck squamous cell carcinoma; MDSC, myeloid-derived suppressor cell; NK, natural killer; TAM, tumor-associated macrophage

HNSCC consists of heterogeneous cell types, each of them plays a role in tumorigenesis including native and disease-causing cells, and in this case, cancer cells as well as other cells within the microenvironment, including immune cells, nonimmune cells, and extracellular components [17–19]. Specifically, immune cells are composed of T cells, B cells, natural killer (NK) cells, myeloid-derived suppressor cells (MDSCs), TAMs, and so on, whereas nonimmune cells mainly consist of CAFs. In addition, biological factors such as bacteria and viruses also affect the tumor ecosystem of HNSCC. Indeed, abiotic factors (e.g., extracellular matrix [ECM], energy, oxygen, and therapeutic interventions) are also key conditions for maintaining the stability of the tumor ecosystem. A summary of components of the HNSCC ecosystem is shown in Fig. 1.

## The interaction of various components in HNSCC

The tumor ecosystem is composed of various living organisms and abiotic factors, which interact with each other and affect the occurrence and development of tumors through different mechanisms.

### Biological factors

#### Cancer stem cells

Cancer stem cells (CSCs) are a subgroup of cells in heterogeneous tumor masses with self-renewal and differentiation capabilities. CSCs play a vital role in tumorigenesis, tumor progression, drug resistance, and maintenance of heterogeneity [20]. The protection of CSCs by the tumor ecosystem is manifested by maintaining their survival and self-renewal ability, helping them resist chemoradiotherapy, and even inducing normal and non-CSCs to transform into CSCs [21, 22]. Epithelial–mesenchymal transition (EMT) has been confirmed as one of the significant mechanisms for the acquisition of a CSC-like phenotype in non-CSCs through transforming growth factor β (TGF-β) [23, 24].

The crosstalk between CSCs and the tumor ecosystem is complex and dynamic. It has been demonstrated that endothelial interleukin 6 (IL-6) enhances the self-renewal of CSCs through the activation of Bmi-1 and downstream signal transducer and activator of transcription 3 (STAT3) signaling in human HNSCC, while humanized anti-IL-6R antibody (tocilizumab) significantly inhibits CSC-mediated tumorigenic ability [25, 26]. The maintenance of some characteristics (e.g., EMT) of CSCs depend on the involvement of CAF-derived factors affecting CSCs and their surrounding immune cells. It was found that TGF-β1, a well-known inducer of EMT, was consistently elevated in nasopharyngeal carcinoma tissue CAFs compared with normal mucosal fibroblasts and dermal fibroblasts [27]. In addition, CSCs can be modulated directly by TAMs, including regulation of sex determining region Y box 2 (SOX2), a transcription factor highly related to stemness [28]. TAM increased the availability of hyaluronic acid, and in turn, increased phosphatidylinositol-3-kinase (PI3K)-eukaryotic translation initiation factor 4E-binding protein (4EBP1)-SOX2 signaling and the CSC fraction by binding to CD44 [29]. Due to the pivotal role of CSCs in tumor ecosystem, the CSC-targeting strategies have become a novel conceptual framework for HNSCC. CSC subpopulation in HNSCC overexpresses α_v_β_5_ which is associated with angiogenesis and lymphangiogenesis [30]. The installation of cyclic Arg-Gly-Asp (cRGD) peptide on cisplatin-loaded nanomedicines can target α_v_β_5_ effectively [31], which is an advantageous method for targeting CSCs in HNSCC. Besides, valproic acid, a histone deacetylase inhibitor, could downregulate the expression of stemness markers (e.g., CD44) of oral cancer stem cells [32]. A Phase II clinical trial about valproic acid in combination with cisplatin and cetuximab in R/M HNSCC is ongoing (NCT02624128).

#### Stromal cells

**Fig. 2:**
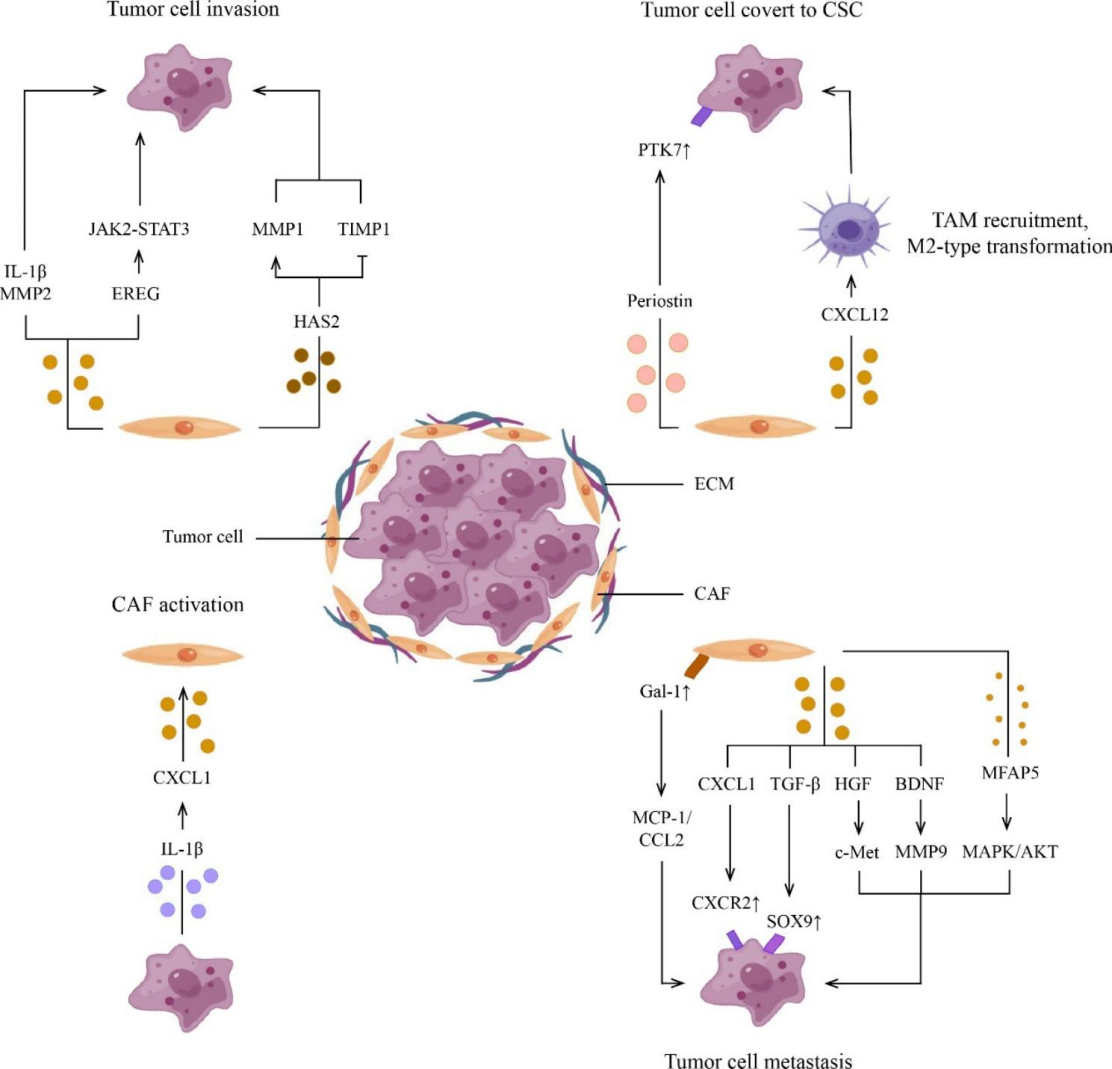
Crosstalk between CAFs and tumor cells in the tumor ecosystem. CAFs can mediate tumor progression and transformation by interacting with tumor cells through secreting multiple chemokines, cytokines, and other effector molecules such as IL-1β, MMP2, EREG. Notably, CAFs can be activated by tumor cells through signals including IL-1β. Abbreviations: BDNF, brain-derived neurotrophic factor; CAF, cancer-associated fibroblast; CCL2, C–C chemokine ligand 2; CSC, cancer stem cell; CXCL12, C-X-C chemokine ligand 12; CXCR2, C-X-C motif chemokine receptor 2; ECM, extracellular matrix; Gal-1, galectin-1; HAS2, hyaluronan synthase 2; EREG, epiregulin; HGF, hepatocyte growth factor; IL-1β, interleukin-1β; JAK2-STAT3, janus kinase 2-signal transducer and activator of transcription 3; MAPK/AKT, mitogen-activated protein kinase/ protein kinase B; MCP-1, monocyte chemoattractant protein-1; c-Met, cellular-mesenchymal epithelial transition factor; MFAP5, microfibrillar associated protein 5; MMP2, matrix metalloproteinase 2; PTK7, protein tyrosine kinase 7; SOX9, sex determining region Y box 9; TGF-β, transforming growth factor-beta; TIMP1, tissue inhibitor matrix metalloproteinase 1

CAFs are the most abundant stromal cells in HNSCC and play a crucial role in tumor angiogenesis, invasion, and metastasis [33, 34]. In the tumor ecosystem, fibroblasts convert to CAFs via TGF-β and IL-1β signaling pathways [35, 36]. The possible CAF subtypes in HNSCC were identified, termed CAF-D and CAF-N [16, 37]. CAF-D (CD44^+^ CD90^+^ α-SMA^high^/BMP4 ^low^) synthesized TGF-β1 that are essential for cancer invasion, whereas CAF-N (CD44^+^ CD90^+^ α-SMA^low^/BMP4 ^high^) included intrinsically motile fibroblasts [37, 38]. There are many avenues that CAFs can take to influence HNSCC tumor cell behaviors, as shown in Fig. 2. Researchers have discovered exosomal microRNAs (miRNAs) and their functional significance [39–42]. Once internalized, CAF-exosomes lead to increased malignant features by altering the respective miRNA levels in recipient cancer cells. CAF-exosomes are rich in miR-196a targeting *ING5* and *CDKN1B* mRNAs, which lead to the reduction of their encoded proteins, respectively, thereby promoting tumor cell proliferation and inhibiting cell apoptosis [40]. CAF-exosomes lack miR-34a-5p and miR-3188, leading to the activation of Axl, and BLC2, thereby facilitating tumor cell migration, invasion, and EMT [41, 42]. In contrast, how tumor cells affect CAFs has been less reported. Wang et al. found that HPV-positive HNSCC-derived exosomal miR-9-5p inhibits TGF-β signaling-mediated phenotypic transformation of fibroblasts through NADPH oxidase 4 (NOX4), reducing CAF infiltration in HNSCC [43]. Other cells within the tumor ecosystem are also affected by the interaction with CAFs. On the one hand, CAFs can induce apoptosis of T cells. CAFs inhibit CD8^+^ T cell function by inducing IL-6 autocrine loops and interacting with T helper 17 (Th17) cells [44]. On the other hand, CAFs alone or in concert with tumor cells differentiate monocytes into the protumor macrophage phenotype that exert an inhibitory effect on T cells by secreting IL-10, TGF-β, and arginase I [45]. In view of malignant features of CAFs, efforts have been paid to turn foes to friends. One of the feasible strategies for CAF-targeting ecotherapy is altering CAF activation or function. Current study found that gold nanoparticles (GNPs) enhanced the expression of lipogenesis genes in CAFs, thereby transforming activated CAFs to a quiescence state [46]. Another study demonstrated that CAF-induced tumor progression was suppressed by TGF-β receptor inhibitor LY2109761. Given the essential role of TGF-β in the activation of CAFs, targeting TGF-β signaling appears to be a potential ecotherapy of HNSCC.

**Fig. 3:**
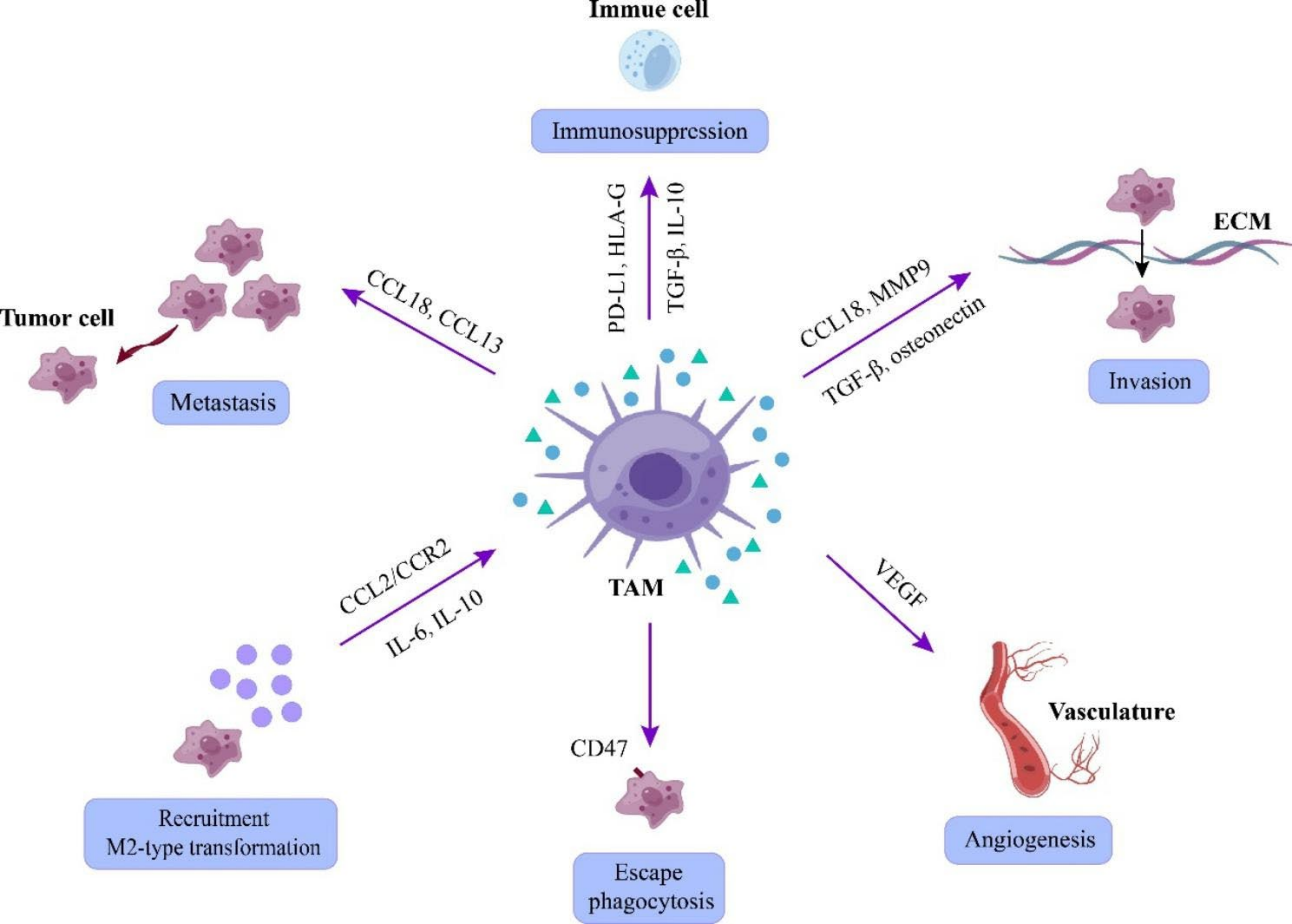
Overview of protumor effects of TAMs in head and neck squamous cell carcinoma. TAMs promote tumor progression by participating in tumor invasion, metastasis, and angiogenesis as well as immunosuppression. Abbreviations: CCL2, C–C chemokine ligand 2; CCR2, CC chemokine receptor 2; ECM, extracellular matrix; HLA-G, human leucocyte antigen-G; IL-6, interleukin-6; MMP9, matrix metalloproteinase 9; PD-L1, programmed death-ligand 1; TAM, tumor-associated macrophage; TGF-β, transforming growth factor-beta; VEGF, vascular endothelial growth factor

TAMs are the most important stromal cell in the tumor ecosystem in HNSCC, and can orchestrate tumor-promoting inflammation [47]. TAMs differ from tissue-resident macrophages in that they are modified in the tumor ecosystem, causing some of them to lose their ability to phagocytose and present tumor antigens to T cells [48, 49]. Activated macrophages are divided into M1 and M2 macrophages. The M1 phenotype promotes Th1 response and displays anti-tumor properties, whereas the M2 phenotype mediates Th2 responses [50]. In HNSCC, tumor cells can recruit and drive macrophages to M2 phenotype by producing C–C chemokine ligand 2 (CCL2)/ CC chemokine receptor 2 (CCR2) [51], IL-6 [52], and IL-10 [51]. TAMs promote tumor progression by producing/expressing a variety of immunosuppressive molecules (e.g.,CCL13 [53], CCL18 [54], matrix metalloproteinase 9 [MMP9], osteonectin [51], TGF-β [55], programmed death-ligand 1 [PD-L1] [56], human leucocyte antigen-G [HLA-G] [45], TGF-β, and IL-10 [57]), and stimulating angiogenesis by producing vascular endothelial growth factor (VEGF) [51] (Fig. 3). CCL18 derived from M2 TAM promotes metastasis by inducing EMT and stemness in HNSCC *in vitro* [54]. Interestingly, cancer cells themselves are also able to produce CCL18, as demonstrated in oral squamous cell carcinoma (OSCC) [58]. In addition, it has been shown that M2 TAM inhibits anti-tumor immunity by downregulating M1 through the secretion of anti-inflammatory cytokines, including IL-10 [59]. Strategies targeting TAM have been thoroughly investigated, including inhibition of macrophage recruitment/survival, re-polarization of TAMs to M1-like status, and recovering macrophage-mediated phagocytosis of cancer cells. CD47 is a “don’t eat me” signal against macrophages, which is elevated in the tumor cells [49]. Up to now, the activity of targeting CD47 monoclonal antibody (mAbs) alone have been demonstrated in solid tumors, and combining with pembrolizumab shows remarkable efficacy [60]. Thus, bispecific antibodies (BsAbs) targeting CD47 is emerging. In HNSCC, PF-07257876 is a CD47-PD-L1 bispecific antibody, and a Phase I clinical trial is underway (NCT04881045). Besides, ALX148, a novel CD47 blocking agent, in combination with pembrolizumab and/or chemotherapy has entered clinical trials (NCT04675333, NCT04675294).

**Fig. 4:**
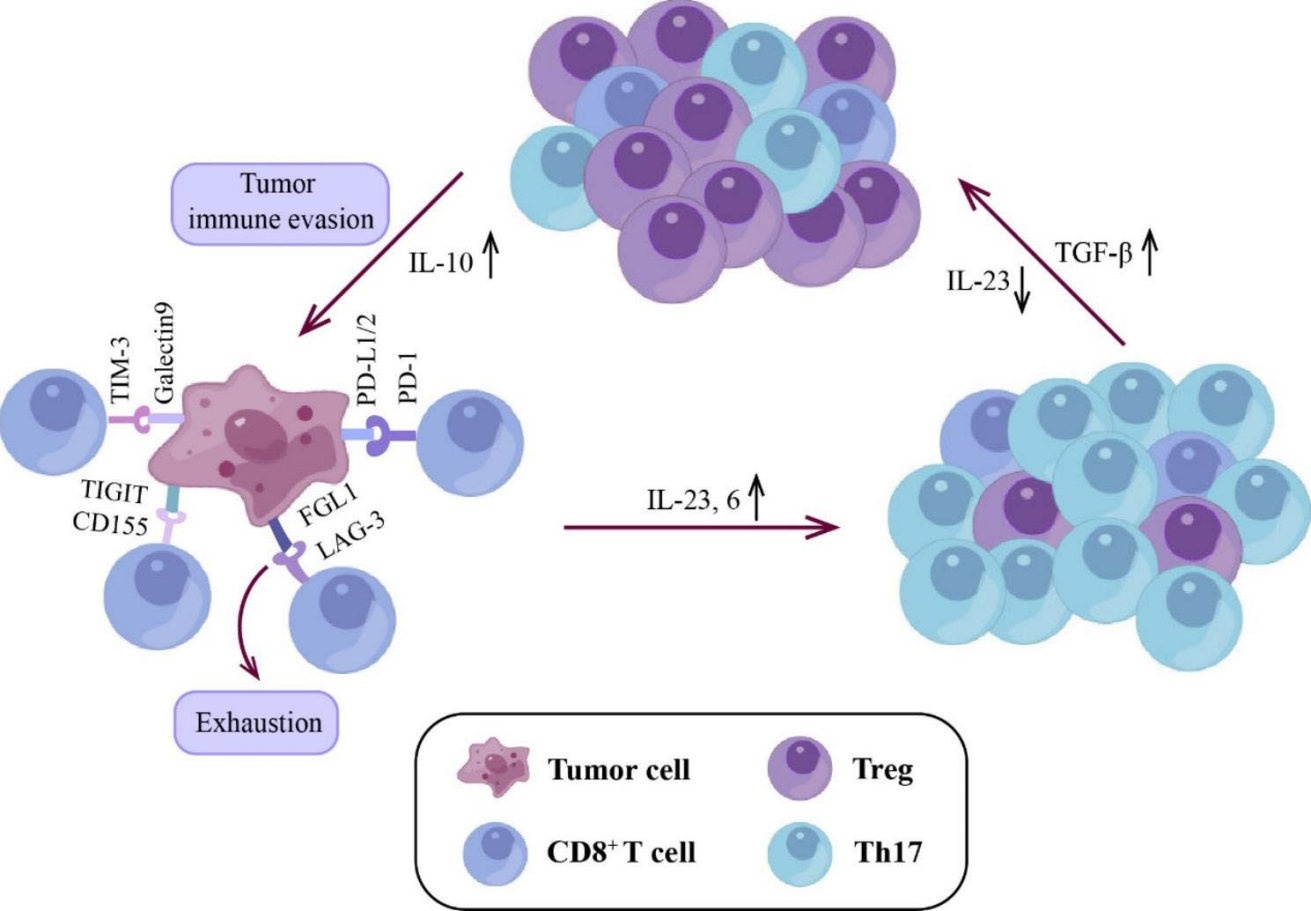
Crosstalk between tumor cells and T cells. IL-23 and -6 secreted by tumor cells recruit Th17, whereas the ratio of Th17 and Tregs influence HNSCC progression. Abbreviations: IL-6, interleukin-6; FGL1, fibrinogen-like protein 1; LAG-3, lymphocyte activated gene-3; PD-1, programmed cell death protein-1; PD-L1/2, programmed death-ligand 1/2; Th17, T helper 17; TIM-3, T cell immunoglobulin mucin-3

T cells are an essential part of the adaptive immune response system. In HNSCC, four sub-clusters of T cells were divided, including two cytotoxic CD8^+^ T cell populations (CD8^+^ T and CD8^+^ T_exhausted_), regulatory T cells (Tregs), and conventional CD4^+^ T helper cells (CD4^+^ T_conv_). Interestingly, higher proportions of Tregs than other T cell subsets were strongly associated with favorable survival outcomes in HNSCC. However, low CD8^+^ T cells were likely correlated with improved survival [9]. Dysfunctional tumor infiltrating lymphocytes (TILs) in HNSCC is characterized by the upregulation of several immune checkpoint markers, such as cytotoxic T lymphocyte antigen-4 (CTLA-4) [61], programmed cell death protein-1 (PD-1) [62], T cell immunoglobulin mucin-3 (TIM-3) [63], lymphocyte activated gene-3 (LAG-3) [64], fibrinogen-like protein 1 (FGL1) [65], Glucocorticoid-induced tumor necrosis factor receptor family-related protein (GITR) [66], and V-domain Ig suppressor of T cell activation (VISTA) [64]. Immune checkpoint blockade is conductive to the heightened functionality of their cytotoxic T cells. The elevated levels of tumor infiltrated Tim-3^+^ Tregs also lead to CD8^+^ T cell dysfunction in HNSCC [63, 67]. In contrast, Th17 cells showed antitumor activity by impairing the proliferation and angiogenesis of HNSCC [67]. The increase in Th17 is mainly due to the secretion of IL-23 and -6 from tumor cells and TILs, and IL-1β released by immune cells in HNSCC [67]. In the late phase of HNSCC, tumor cells promote skew toward Treg owing to the decrease levels of IL-23. Accordingly, a shift in the cytokine milieu from the Th17 sustaining pro-inflammatory IL-23 toward TGF-β may decrease the ratio of Th17 to Tregs [68, 69]. This decrease induces tumor-promoting Treg differentiation and increases production of the anti-inflammatory cytokine IL-10 in HNSCC, which is favorable to cancer progression (Fig. 4) [70]. Currently, rejuvenating exhausted T cell by immune checkpoint inhibitors (ICIs), such as anti-PD-1 and anti-PD-L1 antibodies, show promising effectiveness in solid tumors, which can be expected to improve the prognosis and overall survival in patients with R/M HNSCC. Consequently, strategies for breaking Treg/Th17 balance toward antitumor status, uncovering novel ICIs such as TIGIT, and the combination of ICIs with treatments including RT/chemotherapy would be favorable to HNSCC patients.

B cells are the second batch of adaptive immune cells discovered in the TME and is elevated in HNSCC [71]. The chemokine, C-X-C chemokine ligand 13 (CXCL13), produced by follicular dendritic cells, is important for B cell recruitment in the tumor ecosystem [72]. However, most studies analyzing TILs in HNSCC did not include tumor-associated B cells. Recently, studies have shown that B lymphocytes and their subsets are associated with the positive prognosis of many cancers, including HNSCC [73]. In HPV-positive HNSCC, infiltrations of memory B cell and plasma cell are increased, and high B cell infiltration is associated with better prognosis [74–76]. The main reason is that it can produce IL-10, TGF-β, and IL-35 in anti-tumor immunity [77]. Antibody-secreting cells and germinal center B cells, as well as plasma cells, can also be found in the tumor ecosystem of HPV-positive HNSCC [71]. But it remains unclear to what extent these cells secrete tumor-antigen-specific antibodies which has a direct anti-tumor effect. In ovarian cancer, polyclonal immunoglobulin A (IgA) antibodies derived from tumor-associated B cells bind IgA receptors on the surface of tumor cells, which can result in tumor proliferation inhibition [78]. This finding portends that ecotherapy that augments B cell responses could be an alternative strategy for HNSCC treatment.

MDSCs are the most important protector of the tumor ecosystem, preventing tumor cells from immune surveillance. MDSCs not only produce reactive oxygen species (ROS), but also continuously inhibit T cell activation [79]. They interact with each other to catalyze the nitrification of T cell receptors, thereby inducing T cell tolerance [79]. In HNSCC, elevated MDSC levels have been reported to upregulate inflammatory mediators, such as IL-6, rendering the ecosystem unfavorable for antigen-presenting cell maturation and thus indirectly promoting tumor cell growth [80]. In addition, inhibition of janus kinase 2 (JAK2)/STAT3 was found to reduce MDSCs in the tumor ecosystem through the inhibition of VEGFA and casein kinase 2 (CK2) in HNSCC transgenic mouse models [81]. Elimination of MDSC improves the ability of the host immune system to attack cancer cells and improves the effectiveness of immunotherapy [80]. Interestingly, organs of future metastasis seem to be prepared before the arrival of tumor cells. MDSCs are considered the key determinants of this pre-metastatic niche (PMN) [82]. Expanding experimental evidence indicates that MDSC-derived TGF-β, calprotectin (S100A8/A9), and VEGF promote PMN formation and metastasis by communicating with the immune system, endothelial cells, fibroblasts, and hepatic stellate cells [83], although the exact mechanism in HNSCC remains to be confirmed. Accumulating evidences support that reduction of MDSC level is beneficial to augment anti-tumor immunity. Inhibiting MDSCs trafficking by CXCR1/2 inhibitor significantly enhances NK-cell immunotherapy in head and neck cancer [84]. Of note, a recent clinical study pointed out that β-glucan therapy can disrupt the suppressive function of MDSCs, thereby promoting anti-tumor immunity and increasing recurrence-free survival rate in OSCC patients [85].

Neutrophils are key players in inflammatory cell infiltration in cancer. They can exert anti-tumor and pro-tumor activities, and exhibit unexpected functional plasticity [86]. Like TAMs, it is polarized in different activation states: an anti-tumor N1 or a protumor N2 phenotype. The anti-tumor effect of N1 neutrophils is related to their cytotoxicity and the regulation of anti-tumor immune responses, while N2 neutrophils induced by tumor-derived signals can promote the proliferation, migration, and invasion of tumor cells and mediate immunosuppression [87]. In HNSCC, tumor cells produce macrophage-inhibiting factor, which recruits neutrophils through the engagement of CXCR2 [88]. Another interesting ability of neutrophils is to induce cell death termed NETosis, a process by which neutrophils release cytotoxic molecules shaping a neutrophil extracellular trap (NETs) [89]. NETs have been recognized as the first line of defense to mediate the response of the host [90]. The development of NETs has potential relevance to tumor initiation, progression, recurrence, and metastasis, and plays an essential regulatory role in the tumor ecosystem. NET-related gene signatures are related to prognosis and immunotherapy response of patients with cancers. NIFK, a novel NET-related gene, was found to be significantly upregulated in HNSCC patients that had poor prognoses. The level of NIFK was relevant to regulating cell cycle and DNA replication as well as WNT and p53 signaling pathways [91]. Besides, tumor cells are also able to escape immune surveillance by the NETs [90]. In OSCC, patients in late stages exhibit elevated NET levels compared to early stages [92]. Furthermore, the depletion of IL-6, IL-8, and tumor necrosis factor-alpha (TNF-α) contributes to the reduction of plasma NETs [92]. Currently, the safety and tolerance of HuMax-IL8 as an IL-8 inhibitor has been demonstrated [93]. Given the above knowledge, it is valuable to investigate the effect of IL-8 inhibitors on NETs and tumor progression.

#### Vasculature

Angiogenesis is a biological process that carries oxygen and nutrition to body tissues and organs, and constantly nourishes diseases such as cancer. It is a basic step in the transformation from benign to malignant tumor and is thought to result from the imbalance between pro/anti-angiogenic factors caused by malignant and normal cells [94]. At present, the poor prognosis of HNSCC mainly lies in the incurable R/M diseases, and angiogenesis plays a key role in lymph node infiltration and distant metastasis. Interestingly, there is close molecular crosstalks between tumor cells and endothelial cells in HNSCC. IL-6, CXCL8, and epidermal growth factor (EGF) secreted by tumor cells improve the survival rate and angiogenic potential of endothelial cells through the activation of STAT3/ protein kinase B (AKT)/extracellular signal-regulated kinase (ERK) signaling pathways, respectively [95], while VEGF secreted by endothelial cells can enhance the migration of tumor cells and protect them from apoptosis [96]. Besides, overexpression of TGF-β1 in HNSCC leads to inflammation, angiogenesis, and epithelial hyperproliferation [97]. Immune cells are also linked to angiogenesis. Studies have shown that M2 TAMs mediated mainly by hypoxia-inducible factor-2α (HIF-2α) and VEGF under a hypoxic ecosystem can promote angiogenesis in OSCC [98]. To date, there are an increasing number of pre-clinical and clinical studies evaluating the safety and efficacy of VEGFR-targeted strategies. Axitinib, a potent inhibitor of VEGER, improved 6-month overall survival compared with controls in unresectable R/M HNSCC [99].

#### Virus

HPV has emerged as a risk factor for HNSCC [100]. HPV-16 is the main pathogenic group in over 200 types of HPVs [101]. HPV-16 is an 8 kb virus, which consists of seven early genes (E1-E7) and two late genes (L1 and L2). Early genes are involved in viral genome replication and late genes encode viral capsid proteins [102]. Among them, E6 and E7 are two major oncogenes that lead to malignant transformation. E6 and E7 mediate cell cycle loss, promote uncontrolled cell proliferation, and induce chromosomal instability by degrading tumor suppressor gene p53 and retinoblastoma proteins [103]. HPV-positive oropharyngeal SCC (OPSCC) has a high cure rate with a better prognosis, whereas HPV-negative HNSCC tends to have a non-ideal prognosis [100]. Tumor-infiltrating immune cells ascertain the malignant evolvement of tumors and are a source of significant prognostic factors for patients [104]. A study showed that better prognosis was positively correlated with the infiltration of CD4^+^ T cells, CD8^+^ T cells, follicular helper T cells, and Tregs [105]. Based on HPV status, the extent and features of intratumoral immune cell infiltration in HNSCC patients vary significantly. Compared with HPV-negative tumor tissues, HPV-positive HNSCCs exhibited higher scores for various immune cell types, including plasma cells, basophils, T cells, and B cells [106, 107]. Hence, HPV may influence prognosis by affecting immune cell infiltration. Comparison of tumor-infiltrating immune cells between HPV-positive and HPV-negative HNSCC with normal tissue is listed in Table 2 [108, 109]. Though HPV is an oncogenic factor for HNSCC, HPV-positive HNSCC patients shows better outcomes OS (9.1 months vs. 7.7 months) with anti-PD-1 therapy compared with that in HPV-negative patients [110, 111].

**Table 2:** Changes of major immune cell infiltration compared with normal tissue

Cell types	HPV-positive	HPV-negative
CD4^+^ T cells	↑	↓
Th1 cells	↑	↑
Th2 cells	↑↑	↑↑
Tregs	↑	-
CD8^+^ T cells	↑↑	↑
B cells	↑↑	-
DCs	-	-
NK cells	-	↓
NKT cells	↑↑	↑
Neutrophils	-	-
Monocytes	-	-
Macrophages	↑↑	↑
Plasma cells	↑↑	-

Th1 cell, T helper 1 cell; DC, dendritic cell; NK cell, natural killer cell

#### Microbiota

There are more than 750 kinds of the oral microbiome, including bacteria, protozoa, and fungi [112]. In a healthy state, these species maintain a relative balance of a certain proportion. However, once the oral ecology is dysregulated, this balance is disrupted, which induces tumorigenesis by suppressing immune responses and mediating chronic inflammation [113]. After antibiotic treatment in mice, the tumor formation rate was significantly reduced, and the reconstruction of HNSCC microbes can promote the development of tumors in germ-free mice [114]. Interestingly, another study found that increased oral abundance of commensal *Corynebacterium* and *Kingella* was related to decreased risk of HNSCC [115]. Recently, researchers preformed an overall analysis of the tumor microbiome of 1526 tumors and adjacent normal tissues and found that intratumor bacteria are mainly intracellular and are present in all cancer cells [116]. In OSCC, *Parvimonas*, *Peptoniphilus* and *Fusobacterium* are the dominant genera [117]. Notably, depletion of intratumor bacteria reduced lung metastasis in breast cancers. During metastasis, intratumor bacteria enhanced resistance to blood stress by activating the RhoA-POCK signaling pathway to reorganize the actin cytoskeleton, which contributed to tumor cell survival [118]. Recently, GeoMx digital spatial profiling platform found that intratumoral bacteria-colonized microniches had immunosuppressive effect by excluding CD3^+^ T cells and recruiting neutrophils [117]. Thus, a deeper understanding of these effects may provide avenue for novel ecotherapy options for HNSCC patients. Recent studies found that *Bifidobacterium breve* and exopolysaccharide which were isolated from a new *Bifidobacterium breve* strain displayed anti-tumor effect on HNSCC [119, 120], which is expected to be a new and acceptable therapeutic method in the future.

### Abiotic factors

In addition to biological factors, ECM, energy, and oxygen serve as abiotic factors that also shape the tumor ecosystem. The ECM of HNSCC is a highly heterogeneous network of cross-linked extracellular molecules with a variety of cytokines, intermediate metabolites, nutrients, hormones, and chemokines secreted by tumor and stromal cells [121]. Increased expression of ECM molecules by CAFs elevates tumor stiffness, which activates oncogenic intracellular signaling pathways, such as β-catenin, Akt, PI3K, and focal adhesion kinase (FAK) pathways, and silences tumor suppressor genes, such as phosphatase and tensin homolog [122]. Stiffness exerts behavioral effects on the adjacent tumor cells, affecting cell proliferation, migration, and invasion, and in turn, impacts the metastatic process [123]. In OSCC, scRNA-seq revealed that matrix-producing CAFs were abundant in the prominent stromal compartment of most tumors examined [16]. Collagen I is the most represented protein in the ECM and is often increased in OSCC [124]. Collagen I contains two CYP1 chains (COL1A1) and a single CYP2 chain (COL1A2). COL1A1 binding with the receptor tyrosine kinase discoidin domain receptor 1 activates the proliferation and migration of HNSCC cells [125]. Meanwhile, COL1A2 chain binding with integrin α_v_β_8_ promotes EMT by activating the FAK/mitogen-activated protein kinase (MEK)/ERK signaling pathway, leading to higher tumor cell aggressiveness in OSCC [126]. Fibronectin is another fibrillar ECM molecule and laminins are one of the primary components of the basement membrane (BM) [127]. As the molecular marker of BM degradation, laminin expression can be important for evaluating the aggressiveness of OSCC [128]. Laminin-5 expression is related to high-intensity tumor budding, suggesting that it is associated with the establishment of an invasive phenotype [129]. Of particular interest, laminin-5 and α_6_β_4_ integrin is downregulated by miR-29s to inhibit tumor survival and invasion [130]. HPV-positive HNSCC has corresponding changes in ECM components, such as increased expression of MMPs [131], which promote ECM remodeling and lead to the release of growth factors and active fragments. Of note, 19-Nor-2α-(3-hydroxypropyl)-1α,25(OH)_2_D_3_ (MART-10) as a novel synthesized vitamin D analog can significantly repress HNSCC cells migration and invasion through inhibiting MMP9 activity [132], supporting a potential value of ECM remodeling as an ecotherapy.

To meet the demand for active cell growth, tumor cells need a lot of energy and macromolecules from the extracellular environment. External signals can then be transduced into the cells and the number of core metabolic pathways, including glycolysis, lipid and amino acid anabolism/catabolism, and mitochondrial metabolism, can be selected to support cell survival [133–135]. Cancer cells increase glucose uptake and ferment it into lactic acid even in the presence of abundant oxygen, which is deemed as the Warburg effect [136]. Lactic acid accumulation is a characteristic of the tumor ecosystem. It facilitates polarization of M2-like macrophage, which promotes immune suppression and tumor progression in human HNSCC [137, 138]. HNSCC also has specific metabolic features. For example, compared with adjacent normal tissues, a higher cellular retinoic acid-binding protein expression was detected in tumor tissues [139], which can exert a positive effect on cell proliferation [140]. Compared with concentrations derived from the corresponding primary lesions, glutathione was enriched in metastatic tumors in HNSCC [141], suggesting a possible effect of glutathione metabolism on metastases in HNSCCs.

Oxygen is a pivotal factor in the tumor ecosystem and is essential for maintaining homeostasis and cellular metabolism through the production of adenosine-5’-triphosphate (ATP). Cancer cells and stromal components often have difficulty accessing nutrients and oxygen during tumor development and progression. Hypoxia stimulates complex carcinogenic changes in tissue [142]. Oxygen deprivation in cancerous tissue often results in the formation of dysfunctional blood vessels, leading to tumor growth and invasion [143]. It has been associated with resistance to radiation and decreased immune infiltration in HNSCC, which can lead to a poor prognosis [144]. HIF-1α is a direct response factor to hypoxia that controls the expression of hypoxia-responsive genes to regulate biological functions of tumor cells, such as apoptosis, CSC formation, migration, angiogenesis, and response to chemotherapy and RT [145]. Studies have demonstrated that hypoxia promotes EMT of HNSCC through HIF-1α [146–149]. Overexpressed HIFs were highly related to mortality risk in HNSCC [150].

## Therapy for the tumor ecosystem

Conventional treatment for HNSCC patients mainly includes surgical resection, RT, and chemotherapy. In addition, cetuximab is the main approved drug for targeted therapy, to combine with RT for the treatment of cisplatin-unfit and locally advanced patients or chemotherapy for R/M patients [151–154]. As a chimeric EGFR IgG1 mAb, cetuximab can block EGFR signaling pathway, inducing cell apoptosis, and reduce the production of MMP and VEGF [155]. Unfortunately, due to the tissue diversity and high genetic heterogeneity of HNSCC, the effectiveness of various therapies is limited [156]. Changes in autonomous and involuntary ecosystems affect the effectiveness of antitumor therapy. The development of drug resistance may due to the cancer-centered mutations of tumor cells during the treatment. Thus, the tumor ecosystem helps to identify elements of effective therapeutic value resulting from tumor-host interactions.

For the HNSCC ecosystem, three conventional treatments represent three classic ecologic ways to eradicate tumors. For more aggressive tumors, more powerful strategies are indispensable to warrant further tumor control. In natural ecosystems, the most effective way to kill a species is to alter its biospheres, such as habitat intervention, disruption of matter/energy flow, and introduction of predators. Similarly in the tumor ecosystem, the most effective way to destroy tumor cells may be to change their environment, including anti-angiogenic metabolism modulators and immunotherapy [157]. The drugs currently in clinical trials for the above suggested modalities are summarized in Table 3. Some drugs have been used in other diseases but are still being tested in HNSCC. However, some cells in the tumor ecosystem, such as CAFs, TAMs, and MDSCs, remain poorly characterized, and clinical drugs targeting these cells are yet to be developed. Of note, the presence of immature immune cells in the tumor ecosystem is a turning point in tumor evolution. Therefore, the ability of the immune system to fight cancer can be enhanced by anti-tumor immune cell maturation agents [107].

**Table 3:** Anti-tumor ecological therapy in HNSCC

Target	Drugs	Phase	Patient enrolled	Trial ID
**Inhibit tumor angiogenesis**				
EGFR	BIBW 2992	Phase 2	Metastatic (stage IVc) or recurrent HNC	NCT00514943
VEGFR-1, -2 and -3	Axitinib	Phase 2	Unresectable R/M HNSCC	NCT02762513
EGFR, HER2 and HER4	Poziotinib (HM781-36B)	Phase 2	R/M HNSCC after failure of or unfit for platinum-containing therapy	NCT02216916
-	Endostatins	Phase 3	R/M HNSCC not suitable for operation or radiotherapy	NCT02630264
EGFR	Panitumumab	Phase 2	R/M HNC incurable by surgery or radiotherapy	NCT00446446
EGFR	Zalutumumab	Phase 2	HNSCC incurable with standard therapy	NCT00542308
EGFR	Lapatinib	Phase 2	R/M HNC	NCT00114283
VEGFR	Cediranib	Phase 2	Recurrent or newly diagnosed metastatic HNC	NCT00458978
**Disrupt metabolic homeostasis**				
	Metformin	Early Phase 1	Stage II-IVB HNSCC	NCT02402348
**Anti-tumor immunity**				
PD-1	Cemiplimab-Rwlc	Phase 2	HNSCC after completion of chemotherapy and radiation treatment	NCT04831450
PD-1/PD-L1	Durvalumab	Phase 1	Non-metastatic, suitable for surgical resection, and stage II-IVB oral cavity, stage III-IVB larynx and hypopharynx, or stage III-IVB HPV/p16 negative intermediate-high risk oropharynx HNC	NCT03635164
PD-1	Pembrolizumab	Phase 2	R/M HNSCC	NCT03813836
PD-L1	Atezolizumab	Phase 2	Resectable HNSCC	NCT04939480
PD-L1/ TGF-β	Bintrafusp alfa	Phase 2	Operable and untreated HNSCC	NCT04428047
PD-L1/ CD47	PF-07257876	Phase 1	Advanced or metastatic HNSCC, non-small cell lung cancer, and ovarian cancer	NCT04881045
CD47	Evorpacept in combination with pembrolizumab, cisplatin/carboplatin and 5-FU	Phase 2	Advanced HNSCC	NCT04675333
CD47	Evorpacept in combination with pembrolizumab	Phase 2	Advanced HNSCC	NCT04675294
IL-1β, IL-2, IL-6, IL-8, GM-CSF, INFγ, TNF-α	IRX-2	Phase 2	Untreated and resectable HNSCC	NCT00210470

EGFR, epidermal growth factor receptor; HER2, human epidermal growth factor receptor 2; HNC, head and neck cancer; HNSCC, head and neck squamous cell carcinoma; IL, Interleukin; INFγ, interferon γ; PD-1, programmed cell death protein-1; PD-L1, programmed death-ligand 1; R/M, recurrent or metastatic; TGF-β, transforming growth factor-beta; TNF-α, tumor necrosis factor-α; VEGFR, vascular endothelial growth factor receptor

**Table 4:** Comparison of emerging model for ecosystem research

Model type	Degree of visualization	Cost	Assay duration	Number of cells per experiment	Drug screening throughput
Mouse PDX	Medium	High	Weeks to months	10^5^-10^6^	Low
GEMM	Low	High	Months	-	Low
Zebrafish PDX	High	Low	2–7 days	10^2^-10^3^	High
Organoid	High	Medium	Weeks to months	10^2^-10^3^	Very high

GEMM, genetically engineered mouse model; PDX, patient-derived xenograft

## Emerging model for ecosystem research

An in-depth understanding of the HNSCC tumor ecosystem will provide an opportunity for the development of more reasonable and effective ecological treatment strategies to control tumor metastasis and improve the prognosis of patients. The models of tumor ecosystem have been used to parameterize the complexity of the HNSCC ecosystem. Patient-derived xenografts (PDXs) provide exceptional opportunities to explore the cellular and molecular mechanisms of tumor progression and therapy resistance [158]. Yao et al. revealed biomarkers of cetuximab resistance and clonal architecture changes underlying acquired resistance in HNSCC using a PDX-based study [159]. However, PDXs have several limitations. The tumor ecosystem of HNSCC is constantly changing, which restrains drug response detection in PDXs. Mouse PDX models are not appropriate to study tumor-associated immune cells and immunotherapy due to the use of immunodeficient mice. However, genetically engineered mouse model (GEMM) allows tumor to evolve within an immunocompetent and autochthonous environment, which makes it fit for immune-oncology research, including HNSCC [160]. The tumor ecosystem comprises a large arsenal of immune cells, nonimmune cells, and extracellular components with tumor cells to regulate tumor progression. GEMMs play a pivotal role in studying how the tumor ecosystem shapes a range of stages of tumor evolvement. Besides, zebrafish has many inherent traits (e.g., direct visualization, low cost, short assay duration, facile analysis and quantification, and high accuracy), facilitating tumor ecosystem studies that are difficult to execute with immunocompromised mouse models [161]. Zebrafish xenograft models were used to study metastasis of tumor cells from site of injection to the tail. In HNSCC, the therapeutic effect of anti-cancer drugs was evaluated by assessing the proliferation/migration in a zebrafish metastasis model [162–164]. Organoid cultures are essential for a basic understanding of tumor biology and drug resistance [165]. A recent study suggested that tumor-derived HNSCC organoids have the potential for personalized therapy [166]. As an *in vitro* tool, it is a promising model to select the right therapy for the right patient, albeit costly and time-consuming.

## Conclusion and perspective

In summary, tumor cells dynamically interact with biological and abiotic factors to shape a self-sustainable tumor ecosystem of HNSCC. With the advances in technologies and the surge of big data, more specific characteristics of tumor ecosystem in various dimensions become disclosed. Emerging single-cell RNA-seq/proteomics allow us to identify different cell subpopulations and signals in the tumor ecosystem, and characterize cellular genomic mutations and copy number aberrations, which have powerful implications for the diagnosis and treatment of HNSCC [167, 168]. While single-cell omics uncovers a range of cell subpopulations, the spatial information is limited. Spatial transcriptomics extend our understanding of cellular localization and interaction in the tumor ecosystem [169]. Recent advances in data management technologies, such as artificial intelligence and deep learning, are important for visualizing the dynamics of the tumor ecosystem and predicting cancer treatment [170–173]. Specifically, the deep learning segmentation model designed by convolutional neural networks can automatically segment the tumor, so that more accurate biopsies can be obtained for the research of its ecosystem in OSCC [174].

Nevertheless, research on the tumor ecosystem of HNSCC is still lacking. The molecular mechanisms of the various cells in the tumor ecosystem are not well understood, which makes it difficult to target the ecosystem for therapy in HNSCC. A more detailed comprehension of the pathological mechanisms of how tumor metabolism of HNSCC is engaged in tumorigenesis and tumor maintenance is required. To further explore whether there are other components in the ecosystem of HNSCC and whether they play an essential role in the tumor is an urgent task. With a deeper understanding of the tumor ecosystem, it is tempting to look forward to that a new era of tumor ecotherapy targeting vulnerability of HNSCC will dawn.

## Data Availability

Not applicable.

## Abbreviations

4EBP1: Eukaryotic translation initiation factor 4E-binding protein.
AKT: Protein kinase B.
ATP: Adenosine-5’-triphosphate.
BM: Basement membrane.
CAF: Cancer-associated fibroblast.
CCL16: C–C chemokine ligand 16.
CK2: Casein kinase 2.
CSC: Cancer stem cell.
CTLA-4: Cytotoxic T lymphocyte antigen-4.
CXCL13: C-X-C chemokine ligand 13.
CXCR2: C-X-C motif chemokine receptor 2.
ECM: Extracellular matrix.
EGF: Epidermal growth factor.
EMT: Epithelial–mesenchymal transition.
ERK: Extracellular signal-regulated kinase.
FGFR: fibroblast growth factor receptor.
FGL1: Fibrinogen-like protein 1.
GITR: Glucocorticoid-induced TNF receptor family- related protein.
HIF-2α: Hypoxia-inducible factor-2α.
HNSCC: Head and neck squamous cell carcinoma.
HPV: Human papillomavirus.
IgA: Immunoglobulin A.
IL-6: Interleukin 6.
ICI: immune checkpoint inhibitor.
ITGA6: Integrin subunit alpha 6.
JAK2: Janus kinase 2.
LAG-3: Lymphocyte activated gene-3.
LAMC2: Laminin subunit gamma 2.
MDSC: Myeloid-derived suppressor cell.
mAb: monoclonal antibody.
miRNA: microRNA.
NET: Neutrophil extracellular trap.
NK: Natural killer.
NOX4: NADPH oxidase 4.
OPSCC: HPV-positive oropharyngeal SCC.
OSCC: Oral squamous cell carcinoma.
PD-1: Programmed cell death protein-1.
PDX: Patient-derived xenograft.
PI3K: Phosphatidylinositol-3-kinase.
PMN: Pre-metastatic niche.
R/M: recurrent or metastatic.
RNA-seq: RNA sequencing.
RT: Radiation therapy.
scRNA-seq: Single-cell RNA-seq.
SOX2: Sex determining region Y box 2.
STAT3: Signal transducer and activator of transcription 3.
TAM: Tumor-associated macrophage.
TGF-β: Transforming growth factor β.
Th17: T helper 17.
TIL: Tumor infiltrating lymphocyte.
TIM-3: T cell immunoglobulin mucin-3.
TME: Tumor microenvironment.
TNF-α: Tumor necrosis factor-alpha.
Treg: Regulatory T cell.
VCAM-1: Vascular cell adhesion molecule 1.
VEGF: Vascular endothelial growth factor.
VISTA: V-domain Ig suppressor of T cell activation.

## References

[1] Sung H, Ferlay J, Siegel RL, Laversanne M, Soerjomataram I, Jemal A, Bray F (2021). Global Cancer Statistics 2020: GLOBOCAN estimates of incidence and Mortality Worldwide for 36 cancers in 185 countries. CA Cancer J Clin.

[2] Argiris A, Karamouzis MV, Raben D, Ferris RL (2008). Head and neck cancer. Lancet.

[3] Liu JC, Bhayani M, Kuchta K, Galloway T, Fundakowski C (2019). Patterns of distant metastasis in head and neck cancer at presentation: implications for initial evaluation. Oral Oncol.

[4] Cillo AR, Kürten CHL, Tabib T, Qi Z, Onkar S, Wang T, Liu A, Duvvuri U, Kim S, Soose RJ (2020). Immune Landscape of viral- and carcinogen-driven Head and Neck Cancer. Immunity.

[5] Chow LQM (2020). Head and Neck Cancer. N Engl J Med.

[6] Greaves M, Maley CC (2012). Clonal evolution in cancer. Nature.

[7] Maley CC, Aktipis A, Graham TA, Sottoriva A, Boddy AM, Janiszewska M, Silva AS, Gerlinger M, Yuan Y, Pienta KJ (2017). Classifying the evolutionary and ecological features of neoplasms. Nat Rev Cancer.

[8] Amend SR, Pienta KJ (2015). Ecology meets cancer biology: the cancer swamp promotes the lethal cancer phenotype. Oncotarget.

[9] Qi Z, Liu Y, Mints M, Mullins R, Sample R, Law T, Barrett T, Mazul AL, Jackson RS, Kang SY et al. Single-Cell Deconvolution of Head and Neck Squamous Cell Carcinoma.Cancers (Basel)2021,13. 10.3390/cancers13061230 PMC7999850 33799782

[10] Wang Y, Mashock M, Tong Z, Mu X, Chen H, Zhou X, Zhang H, Zhao G, Liu B, Li X (2020). Changing Technologies of RNA sequencing and their applications in clinical oncology. Front Oncol.

[11] Li H, Courtois ET, Sengupta D, Tan Y, Chen KH, Goh JJL, Kong SL, Chua C, Hon LK, Tan WS (2017). Reference component analysis of single-cell transcriptomes elucidates cellular heterogeneity in human colorectal tumors. Nat Genet.

[12] Wagner J, Rapsomaniki MA, Chevrier S, Anzeneder T, Langwieder C, Dykgers A, Rees M, Ramaswamy A, Muenst S, Soysal SD (2019). A single-cell atlas of the Tumor and Immune Ecosystem of human breast Cancer. Cell.

[13] Ma L, Hernandez MO, Zhao Y, Mehta M, Tran B, Kelly M, Rae Z, Hernandez JM, Davis JL, Martin SP (2019). Tumor Cell Biodiversity drives Microenvironmental Reprogramming in Liver Cancer. Cancer Cell.

[14] van Galen P, Hovestadt V, Wadsworth Ii MH, Hughes TK, Griffin GK, Battaglia S, Verga JA, Stephansky J, Pastika TJ, Lombardi Story J (2019). Single-cell RNA-Seq reveals AML Hierarchies relevant to Disease Progression and Immunity. Cell.

[15] Levitin HM, Yuan J, Sims PA (2018). Single-cell transcriptomic analysis of Tumor Heterogeneity. Trends Cancer.

[16] Puram SV, Tirosh I, Parikh AS, Patel AP, Yizhak K, Gillespie S, Rodman C, Luo CL, Mroz EA, Emerick KS (2017). Single-cell transcriptomic analysis of primary and metastatic Tumor Ecosystems in Head and Neck Cancer. Cell.

[17] Davis RJ, Van Waes C, Allen CT (2016). Overcoming barriers to effective immunotherapy: MDSCs, TAMs, and Tregs as mediators of the immunosuppressive microenvironment in head and neck cancer. Oral Oncol.

[18] Palmer C, Mulligan JK, Smith SE, Atkinson C (2017). The role of regulatory T cells in the regulation of upper airway inflammation. Am J Rhinol Allergy.

[19] Schulz A, Büttner R, Hagel C, Baader SL, Kluwe L, Salamon J, Mautner VF, Mindos T, Parkinson DB, Gehlhausen JR (2016). The importance of nerve microenvironment for schwannoma development. Acta Neuropathol.

[20] Prager BC, Xie Q, Bao S, Rich JN (2019). Cancer Stem cells: the Architects of the Tumor Ecosystem. Cell Stem Cell.

[21] Walcher L, Kistenmacher AK, Suo H, Kitte R, Dluczek S, Strauß A, Blaudszun AR, Yevsa T, Fricke S, Kossatz-Boehlert U (2020). Cancer Stem Cells-Origins and biomarkers: perspectives for targeted personalized therapies. Front Immunol.

[22] Vengoji R, Ponnusamy MP, Rachagani S, Mahapatra S, Batra SK, Shonka N, Macha MA (2019). Novel therapies hijack the blood-brain barrier to eradicate glioblastoma cancer stem cells. Carcinogenesis.

[23] Chen C, Okita Y, Watanabe Y, Abe F, Fikry MA, Ichikawa Y, Suzuki H, Shibuya A, Kato M (2018). Glycoprotein nmb is exposed on the surface of dormant breast Cancer cells and induces stem cell-like Properties. Cancer Res.

[24] Nakano M, Kikushige Y, Miyawaki K, Kunisaki Y, Mizuno S, Takenaka K, Tamura S, Okumura Y, Ito M, Ariyama H (2019). Dedifferentiation process driven by TGF-beta signaling enhances stem cell properties in human colorectal cancer. Oncogene.

[25] Krishnamurthy S, Warner KA, Dong Z, Imai A, Nör C, Ward BB, Helman JI, Taichman RS, Bellile EL, McCauley LK (2014). Endothelial interleukin-6 defines the tumorigenic potential of primary human cancer stem cells. Stem Cells.

[26] Herzog AE, Warner KA, Zhang Z, Bellile E, Bhagat MA, Castilho RM, Wolf GT, Polverini PJ, Pearson AT, Nör JE (2021). The IL-6R and Bmi-1 axis controls self-renewal and chemoresistance of head and neck cancer stem cells. Cell Death Dis.

[27] Rosenthal E, McCrory A, Talbert M, Young G, Murphy-Ullrich J, Gladson C (2004). Elevated expression of TGF-beta1 in head and neck cancer-associated fibroblasts. Mol Carcinog.

[28] Siegle JM, Basin A, Sastre-Perona A, Yonekubo Y, Brown J, Sennett R, Rendl M, Tsirigos A, Carucci JA, Schober M (2014). SOX2 is a cancer-specific regulator of tumour initiating potential in cutaneous squamous cell carcinoma. Nat Commun.

[29] Gomez KE, Wu F, Keysar SB, Morton JJ, Miller B, Chimed TS, Le PN, Nieto C, Chowdhury FN, Tyagi A (2020). Cancer Cell CD44 mediates Macrophage/Monocyte-Driven regulation of Head and Neck Cancer Stem cells. Cancer Res.

[30] Cedra S, Wiegand S, Kolb M, Dietz A, Wichmann G. Reduced Cytokine Release in Ex Vivo Response to Cilengitide and Cetuximab Is a Marker for Improved Survival of Head and Neck Cancer Patients.Cancers (Basel)2017,9. 10.3390/cancers9090117 PMC5615332 28872582

[31] Miyano K, Cabral H, Miura Y, Matsumoto Y, Mochida Y, Kinoh H, Iwata C, Nagano O, Saya H, Nishiyama N (2017). cRGD peptide installation on cisplatin-loaded nanomedicines enhances efficacy against locally advanced head and neck squamous cell carcinoma bearing cancer stem-like cells. J Control Release.

[32] Lee SH, Nam HJ, Kang HJ, Samuels TL, Johnston N, Lim YC (2015). Valproic acid suppresses the self-renewal and proliferation of head and neck cancer stem cells. Oncol Rep.

[33] De Palma M, Biziato D, Petrova TV (2017). Microenvironmental regulation of tumour angiogenesis. Nat Rev Cancer.

[34] Takahashi H, Sakakura K, Kawabata-Iwakawa R, Rokudai S, Toyoda M, Nishiyama M, Chikamatsu K (2015). Immunosuppressive activity of cancer-associated fibroblasts in head and neck squamous cell carcinoma. Cancer Immunol Immunother.

[35] Dudás J, Fullár A, Bitsche M, Schartinger V, Kovalszky I, Sprinzl GM, Riechelmann H (2011). Tumor-produced, active interleukin-1β regulates gene expression in carcinoma-associated fibroblasts. Exp Cell Res.

[36] Mito I, Takahashi H, Kawabata-Iwakawa R, Horikawa M, Ida S, Tada H, Matsuyama T, Misawa K, Takeda S, Chikamatsu K (2023). Tumor-derived exosomes elicit cancer-associated fibroblasts shaping inflammatory tumor microenvironment in head and neck squamous cell carcinoma. Oral Oncol.

[37] Patel AK, Vipparthi K, Thatikonda V, Arun I, Bhattacharjee S, Sharan R, Arun P, Singh S (2018). A subtype of cancer-associated fibroblasts with lower expression of alpha-smooth muscle actin suppresses stemness through BMP4 in oral carcinoma. Oncogenesis.

[38] Costea DE, Hills A, Osman AH, Thurlow J, Kalna G, Huang X, Pena Murillo C, Parajuli H, Suliman S, Kulasekara KK (2013). Identification of two distinct carcinoma-associated fibroblast subtypes with differential tumor-promoting abilities in oral squamous cell carcinoma. Cancer Res.

[39] Sun LP, Xu K, Cui J, Yuan DY, Zou B, Li J, Liu JL, Li KY, Meng Z, Zhang B (2019). Cancer–associated fibroblast–derived exosomal miR–382–5p promotes the migration and invasion of oral squamous cell carcinoma. Oncol Rep.

[40] Qin X, Guo H, Wang X, Zhu X, Yan M, Wang X, Xu Q, Shi J, Lu E, Chen W, Zhang J (2019). Exosomal miR-196a derived from cancer-associated fibroblasts confers cisplatin resistance in head and neck cancer through targeting CDKN1B and ING5. Genome Biol.

[41] Wang X, Qin X, Yan M, Shi J, Xu Q, Li Z, Yang W, Zhang J, Chen W (2019). Loss of exosomal miR-3188 in cancer-associated fibroblasts contributes to HNC progression. J Exp Clin Cancer Res.

[42] Li YY, Tao YW, Gao S, Li P, Zheng JM, Zhang SE, Liang J, Zhang Y. Cancer-associated fibroblasts contribute to oral cancer cells proliferation and metastasis via exosome-mediated paracrine miR-34a-5p. *EBioMedicine* 2018, 36:209–220. 10.1016/j.ebiom.2018.09.006 PMC6197737 30243489

[43] Wang B, Zhang S, Tong F, Wang Y, Wei L (2022). HPV(+) HNSCC-derived exosomal mir-9-5p inhibits TGF-β signaling-mediated fibroblast phenotypic transformation through NOX4. Cancer Sci.

[44] Fang Y, Chen M, Li G, Yang Y, He P, Chen J, Cheng L, Wu H (2022). Cancer-associated fibroblast-like fibroblasts in vocal fold leukoplakia suppress CD8(+)T cell functions by inducing IL-6 autocrine loop and interacting with Th17 cells. Cancer Lett.

[45] Takahashi H, Sakakura K, Kudo T, Toyoda M, Kaira K, Oyama T, Chikamatsu K (2017). Cancer-associated fibroblasts promote an immunosuppressive microenvironment through the induction and accumulation of protumoral macrophages. Oncotarget.

[46] Hossen MN, Rao G, Dey A, Robertson JD, Bhattacharya R, Mukherjee P (2019). Gold nanoparticle transforms activated Cancer-Associated fibroblasts to quiescence. ACS Appl Mater Interfaces.

[47] Mao L, Zhao ZL, Yu GT, Wu L, Deng WW, Li YC, Liu JF, Bu LL, Liu B, Kulkarni AB (2018). γ-Secretase inhibitor reduces immunosuppressive cells and enhances tumour immunity in head and neck squamous cell carcinoma. Int J Cancer.

[48] Lewis CE, Pollard JW (2006). Distinct role of macrophages in different tumor microenvironments. Cancer Res.

[49] Feng Q, Ma X, Cheng K, Liu G, Li Y, Yue Y, Liang J, Zhang L, Zhang T, Wang X (2022). Engineered bacterial outer membrane vesicles as controllable two-way adaptors to activate macrophage phagocytosis for Improved Tumor Immunotherapy. Adv Mater.

[50] Kim J, Bae JS. Tumor-Associated Macrophages and Neutrophils in Tumor Microenvironment. *Mediators Inflamm* 2016, 2016:6058147. 10.1155/2016/6058147 PMC4757693 26966341

[51] Li B, Ren M, Zhou X, Han Q, Cheng L (2020). Targeting tumor-associated macrophages in head and neck squamous cell carcinoma. Oral Oncol.

[52] Yin X, Han S, Song C, Zou H, Wei Z, Xu W, Ran J, Tang C, Wang Y, Cai Y (2019). Metformin enhances gefitinib efficacy by interfering with interactions between tumor-associated macrophages and head and neck squamous cell carcinoma cells. Cell Oncol (Dordr).

[53] Liu Z, Rui T, Lin Z, Xie S, Zhou B, Fu M, Mai L, Zhu C, Wu G, Wang Y. Tumor-Associated Macrophages Promote Metastasis of Oral Squamous Cell Carcinoma via CCL13 Regulated by Stress Granule.Cancers (Basel)2022,14. 10.3390/cancers14205081 PMC9657876 36291863

[54] She L, Qin Y, Wang J, Liu C, Zhu G, Li G, Wei M, Chen C, Liu G, Zhang D (2018). Tumor-associated macrophages derived CCL18 promotes metastasis in squamous cell carcinoma of the head and neck. Cancer Cell Int.

[55] Maldonado LAG, Nascimento CR, Rodrigues Fernandes NA, Silva ALP, D’Silva NJ, Rossa C (2022). Influence of tumor cell-derived TGF-β on macrophage phenotype and macrophage-mediated tumor cell invasion. Int J Biochem Cell Biol.

[56] Chen SMY, Popolizio V, Woolaver RA, Ge H, Krinsky AL, John J, Danis E, Ke Y, Kramer Y, Bian L (2022). Differential responses to immune checkpoint inhibitor dictated by pre-existing differential immune profiles in squamous cell carcinomas caused by same initial oncogenic drivers. J Exp Clin Cancer Res.

[57] Zhang P, Zhang Y, Wang L, Lou W (2021). Tumor-regulated macrophage type 2 differentiation promotes immunosuppression in laryngeal squamous cell carcinoma. Life Sci.

[58] Jiang X, Liu J, Li S, Jia B, Huang Z, Shen J, Luo H, Zhao J (2020). CCL18-induced LINC00319 promotes proliferation and metastasis in oral squamous cell carcinoma via the miR-199a-5p/FZD4 axis. Cell Death Dis.

[59] Sica A, Mantovani A (2012). Macrophage plasticity and polarization: in vivo veritas. J Clin Invest.

[60] Lakhani NJ, Chow LQM, Gainor JF, LoRusso P, Lee KW, Chung HC, Lee J, Bang YJ, Hodi FS, Kim WS (2021). Evorpacept alone and in combination with pembrolizumab or trastuzumab in patients with advanced solid tumours (ASPEN-01): a first-in-human, open-label, multicentre, phase 1 dose-escalation and dose-expansion study. Lancet Oncol.

[61] Dorta-Estremera S, Hegde VL, Slay RB, Sun R, Yanamandra AV, Nicholas C, Nookala S, Sierra G, Curran MA, Sastry KJ (2019). Targeting interferon signaling and CTLA-4 enhance the therapeutic efficacy of anti-PD-1 immunotherapy in preclinical model of HPV(+) oral cancer. J Immunother Cancer.

[62] Ferris RL, Blumenschein G, Fayette J, Guigay J, Colevas AD, Licitra L, Harrington K, Kasper S, Vokes EE, Even C (2016). Nivolumab for recurrent squamous-cell carcinoma of the Head and Neck. N Engl J Med.

[63] Liu Z, McMichael EL, Shayan G, Li J, Chen K, Srivastava R, Kane LP, Lu B, Ferris RL (2018). Novel Effector phenotype of Tim-3(+) Regulatory T cells leads to enhanced suppressive function in Head and Neck Cancer Patients. Clin Cancer Res.

[64] Wuerdemann N, Pütz K, Eckel H, Jain R, Wittekindt C, Huebbers CU, Sharma SJ, Langer C, Gattenlöhner S, Büttner R et al. LAG-3, TIM-3 and VISTA Expression on Tumor-Infiltrating Lymphocytes in Oropharyngeal Squamous Cell Carcinoma-Potential Biomarkers for Targeted Therapy Concepts.Int J Mol Sci2020,22. 10.3390/ijms22010379 PMC7796181 33396515

[65] Wang J, Sanmamed MF, Datar I, Su TT, Ji L, Sun J, Chen L, Chen Y, Zhu G, Yin W (2019). Fibrinogen-like protein 1 is a major Immune Inhibitory ligand of LAG-3. Cell.

[66] von Witzleben A, Fehn A, Grages A, Ezić J, Jeske SS, Puntigam LK, Brunner C, Kraus JM, Kestler HA, Doescher J (2021). Prospective longitudinal study of immune checkpoint molecule (ICM) expression in immune cell subsets during curative conventional therapy of head and neck squamous cell carcinoma (HNSCC). Int J Cancer.

[67] Kesselring R, Thiel A, Pries R, Trenkle T, Wollenberg B (2010). Human Th17 cells can be induced through head and neck cancer and have a functional impact on HNSCC development. Br J Cancer.

[68] Maggioni D, Pignataro L, Garavello W (2017). T-helper and T-regulatory cells modulation in head and neck squamous cell carcinoma. Oncoimmunology.

[69] Woodford D, Johnson SD, De Costa AM, Young MR. An Inflammatory Cytokine Milieu is Prominent in Premalignant Oral Lesions, but Subsides when Lesions Progress to Squamous Cell Carcinoma.J Clin Cell Immunol2014, 5. 10.4172/2155-9899.1000230 PMC4240319 25419481

[70] Yu L, Yang F, Zhang F, Guo D, Li L, Wang X, Liang T, Wang J, Cai Z, Jin H (2018). CD69 enhances immunosuppressive function of regulatory T-cells and attenuates colitis by prompting IL-10 production. Cell Death Dis.

[71] Wieland A, Patel MR, Cardenas MA, Eberhardt CS, Hudson WH, Obeng RC, Griffith CC, Wang X, Chen ZG, Kissick HT (2021). Defining HPV-specific B cell responses in patients with head and neck cancer. Nature.

[72] Cosgrove J, Novkovic M, Albrecht S, Pikor NB, Zhou Z, Onder L, Mörbe U, Cupovic J, Miller H, Alden K (2020). B cell zone reticular cell microenvironments shape CXCL13 gradient formation. Nat Commun.

[73] Ruffin AT, Cillo AR, Tabib T, Liu A, Onkar S, Kunning SR, Lampenfeld C, Atiya HI, Abecassis I, Kürten CHL (2021). B cell signatures and tertiary lymphoid structures contribute to outcome in head and neck squamous cell carcinoma. Nat Commun.

[74] Zhang S, Wang B, Ma F, Tong F, Yan B, Liu T, Xie H, Song L, Yu S, Wei L (2021). Characteristics of B lymphocyte infiltration in HPV(+) head and neck squamous cell carcinoma. Cancer Sci.

[75] Lechner A, Schlößer HA, Thelen M, Wennhold K, Rothschild SI, Gilles R, Quaas A, Siefer OG, Huebbers CU, Cukuroglu E (2019). Tumor-associated B cells and humoral immune response in head and neck squamous cell carcinoma. Oncoimmunology.

[76] Chen X, Yan B, Lou H, Shen Z, Tong F, Zhai A, Wei L, Zhang F (2018). Immunological network analysis in HPV associated head and neck squamous cancer and implications for disease prognosis. Mol Immunol.

[77] Yuen GJ, Demissie E, Pillai S (2016). B lymphocytes and cancer: a love-hate relationship. Trends Cancer.

[78] Biswas S, Mandal G, Payne KK, Anadon CM, Gatenbee CD, Chaurio RA, Costich TL, Moran C, Harro CM, Rigolizzo KE (2021). IgA transcytosis and antigen recognition govern ovarian cancer immunity. Nature.

[79] Feng S, Cheng X, Zhang L, Lu X, Chaudhary S, Teng R, Frederickson C, Champion MM, Zhao R, Cheng L (2018). Myeloid-derived suppressor cells inhibit T cell activation through nitrating LCK in mouse cancers. Proc Natl Acad Sci U S A.

[80] Tesi RJ (2019). MDSC; the most important cell you have never heard of. Trends Pharmacol Sci.

[81] Liu JF, Deng WW, Chen L, Li YC, Wu L, Ma SR, Zhang WF, Bu LL, Sun ZJ (2018). Inhibition of JAK2/STAT3 reduces tumor-induced angiogenesis and myeloid-derived suppressor cells in head and neck cancer. Mol Carcinog.

[82] Peinado H, Zhang H, Matei IR, Costa-Silva B, Hoshino A, Rodrigues G, Psaila B, Kaplan RN, Bromberg JF, Kang Y (2017). Pre-metastatic niches: organ-specific homes for metastases. Nat Rev Cancer.

[83] Wang Y, Ding Y, Guo N, Wang S (2019). MDSCs: key criminals of Tumor pre-metastatic niche formation. Front Immunol.

[84] Greene S, Robbins Y, Mydlarz WK, Huynh AP, Schmitt NC, Friedman J, Horn LA, Palena C, Schlom J, Maeda DY (2020). Inhibition of MDSC trafficking with SX-682, a CXCR1/2 inhibitor, enhances NK-Cell Immunotherapy in Head and Neck Cancer Models. Clin Cancer Res.

[85] Lo YW, Lee AY, Liu YC, Ko HH, Peng HH, Lee HC, Pan PY, Chiang CP, Cheng SJ (2022). β-glucan therapy converts the inhibition of myeloid-derived suppressor cells in oral cancer patients. Oral Dis.

[86] Piccard H, Muschel RJ, Opdenakker G (2012). On the dual roles and polarized phenotypes of neutrophils in tumor development and progression. Crit Rev Oncol Hematol.

[87] Dumitru CA, Lang S, Brandau S (2013). Modulation of neutrophil granulocytes in the tumor microenvironment: mechanisms and consequences for tumor progression. Semin Cancer Biol.

[88] Dumitru CA, Gholaman H, Trellakis S, Bruderek K, Dominas N, Gu X, Bankfalvi A, Whiteside TL, Lang S, Brandau S (2011). Tumor-derived macrophage migration inhibitory factor modulates the biology of head and neck cancer cells via neutrophil activation. Int J Cancer.

[89] de Bont CM, Boelens WC, Pruijn GJM (2019). NETosis, complement, and coagulation: a triangular relationship. Cell Mol Immunol.

[90] Papayannopoulos V (2018). Neutrophil extracellular traps in immunity and disease. Nat Rev Immunol.

[91] Li Q, Chen W, Li Q, Mao J, Chen X (2022). A novel neutrophil extracellular trap signature to predict prognosis and immunotherapy response in head and neck squamous cell carcinoma. Front Immunol.

[92] Li B, Liu Y, Hu T, Zhang Y, Zhang C, Li T, Wang C, Dong Z, Novakovic VA, Hu T, Shi J (2019). Neutrophil extracellular traps enhance procoagulant activity in patients with oral squamous cell carcinoma. J Cancer Res Clin Oncol.

[93] Bilusic M, Heery CR, Collins JM, Donahue RN, Palena C, Madan RA, Karzai F, Marté JL, Strauss J, Gatti-Mays ME (2019). Phase I trial of HuMax-IL8 (BMS-986253), an anti-IL-8 monoclonal antibody, in patients with metastatic or unresectable solid tumors. J Immunother Cancer.

[94] Pulkkinen HH, Kiema M, Lappalainen JP, Toropainen A, Beter M, Tirronen A, Holappa L, Niskanen H, Kaikkonen MU, Ylä-Herttuala S, Laakkonen JP (2021). BMP6/TAZ-Hippo signaling modulates angiogenesis and endothelial cell response to VEGF. Angiogenesis.

[95] Neiva KG, Zhang Z, Miyazawa M, Warner KA, Karl E, Nör JE (2009). Cross talk initiated by endothelial cells enhances migration and inhibits anoikis of squamous cell carcinoma cells through STAT3/Akt/ERK signaling. Neoplasia.

[96] Shigetomi S, Imanishi Y, Shibata K, Sakai N, Sakamoto K, Fujii R, Habu N, Otsuka K, Sato Y, Watanabe Y (2018). VEGF-C/Flt-4 axis in tumor cells contributes to the progression of oral squamous cell carcinoma via upregulating VEGF-C itself and contactin-1 in an autocrine manner. Am J Cancer Res.

[97] Lu SL, Reh D, Li AG, Woods J, Corless CL, Kulesz-Martin M, Wang XJ (2004). Overexpression of transforming growth factor beta1 in head and neck epithelia results in inflammation, angiogenesis, and epithelial hyperproliferation. Cancer Res.

[98] Teixeira LR, Almeida LY, Silva RN, Mesquita ATM, Colturato CBN, Silveira HA, Duarte A, Ribeiro-Silva A, León JE (2019). Young and elderly oral squamous cell carcinoma patients present similar angiogenic profile and predominance of M2 macrophages: comparative immunohistochemical study. Head Neck.

[99] Swiecicki PL, Bellile EL, Brummel CV, Brenner JC, Worden FP (2021). Efficacy of axitinib in metastatic head and neck cancer with novel radiographic response criteria. Cancer.

[100] Sabatini ME, Chiocca S (2020). Human papillomavirus as a driver of head and neck cancers. Br J Cancer.

[101] Ferreiro-Iglesias A, McKay JD, Brenner N, Virani S, Lesseur C, Gaborieau V, Ness AR, Hung RJ, Liu G, Diergaarde B (2021). Germline determinants of humoral immune response to HPV-16 protect against oropharyngeal cancer. Nat Commun.

[102] Berman TA, Schiller JT (2017). Human papillomavirus in cervical cancer and oropharyngeal cancer: one cause, two diseases. Cancer.

[103] de Bakker T, Journe F, Descamps G, Saussez S, Dragan T, Ghanem G, Krayem M, Van Gestel D (2021). Restoring p53 function in Head and Neck squamous cell carcinoma to improve treatments. Front Oncol.

[104] Solomon B, Young RJ, Rischin D (2018). Head and neck squamous cell carcinoma: Genomics and emerging biomarkers for immunomodulatory cancer treatments. Semin Cancer Biol.

[105] Yao Y, Yan Z, Lian S, Wei L, Zhou C, Feng D, Zhang Y, Yang J, Li M, Chen Y. Prognostic value of novel immune-related genomic biomarkers identified in head and neck squamous cell carcinoma.J Immunother Cancer2020,8. 10.1136/jitc-2019-000444 PMC7390201 32719094

[106] Partlová S, Bouček J, Kloudová K, Lukešová E, Zábrodský M, Grega M, Fučíková J, Truxová I, Tachezy R, Špíšek R, Fialová A (2015). Distinct patterns of intratumoral immune cell infiltrates in patients with HPV-associated compared to non-virally induced head and neck squamous cell carcinoma. Oncoimmunology.

[107] Mortezaee K, Majidpoor J (2021). (Im)maturity in Tumor Ecosystem. Front Oncol.

[108] Zhou D, Wang J, Wang J, Liu X (2021). Profiles of immune cell infiltration and immune-related genes in the tumor microenvironment of HNSCC with or without HPV infection. Am J Transl Res.

[109] Mito I, Takahashi H, Kawabata-Iwakawa R, Ida S, Tada H, Chikamatsu K (2021). Comprehensive analysis of immune cell enrichment in the tumor microenvironment of head and neck squamous cell carcinoma. Sci Rep.

[110] Xu Y, Zhu G, Maroun CA, Wu IXY, Huang D, Seiwert TY, Liu Y, Mandal R, Zhang X (2021). Programmed Death-1/Programmed death-ligand 1-Axis Blockade in recurrent or metastatic Head and Neck squamous cell Carcinoma Stratified by Human Papillomavirus Status: a systematic review and Meta-analysis. Front Immunol.

[111] Ferris RL, Blumenschein G, Fayette J, Guigay J, Colevas AD, Licitra L, Harrington KJ, Kasper S, Vokes EE, Even C (2018). Nivolumab vs investigator’s choice in recurrent or metastatic squamous cell carcinoma of the head and neck: 2-year long-term survival update of CheckMate 141 with analyses by tumor PD-L1 expression. Oral Oncol.

[112] Mosaddad SA, Tahmasebi E, Yazdanian A, Rezvani MB, Seifalian A, Yazdanian M, Tebyanian H (2019). Oral microbial biofilms: an update. Eur J Clin Microbiol Infect Dis.

[113] Irfan M, Delgado RZR, Frias-Lopez J (2020). The oral Microbiome and Cancer. Front Immunol.

[114] Frank DN, Qiu Y, Cao Y, Zhang S, Lu L, Kofonow JM, Robertson CE, Liu Y, Wang H, Levens CL (2022). A dysbiotic microbiome promotes head and neck squamous cell carcinoma. Oncogene.

[115] Hayes RB, Ahn J, Fan X, Peters BA, Ma Y, Yang L, Agalliu I, Burk RD, Ganly I, Purdue MP (2018). Association of oral Microbiome with Risk for Incident Head and Neck squamous cell Cancer. JAMA Oncol.

[116] Nejman D, Livyatan I, Fuks G, Gavert N, Zwang Y, Geller LT, Rotter-Maskowitz A, Weiser R, Mallel G, Gigi E (2020). The human tumor microbiome is composed of tumor type-specific intracellular bacteria. Science.

[117] Galeano Niño JL, Wu H, LaCourse KD, Kempchinsky AG, Baryiames A, Barber B, Futran N, Houlton J, Sather C, Sicinska E (2022). Effect of the intratumoral microbiota on spatial and cellular heterogeneity in cancer. Nature.

[118] Fu A, Yao B, Dong T, Chen Y, Yao J, Liu Y, Li H, Bai H, Liu X, Zhang Y (2022). Tumor-resident intracellular microbiota promotes metastatic colonization in breast cancer. Cell.

[119] Wang L, Wang Y, Li Q, Tian K, Xu L, Liu G, Guo C (2019). Exopolysaccharide, isolated from a novel strain Bifidobacterium breve lw01 possess an Anticancer Effect on Head and Neck Cancer - genetic and biochemical evidences. Front Microbiol.

[120] Wang L, Vuletic I, Deng D, Crielaard W, Xie Z, Zhou K, Zhang J, Sun H, Ren Q, Guo C (2017). Bifidobacterium breve as a delivery vector of IL-24 gene therapy for head and neck squamous cell carcinoma in vivo. Gene Ther.

[121] Denton AE, Roberts EW, Fearon DT (2018). Stromal cells in the Tumor Microenvironment. Adv Exp Med Biol.

[122] Peltanova B, Raudenska M, Masarik M (2019). Effect of tumor microenvironment on pathogenesis of the head and neck squamous cell carcinoma: a systematic review. Mol Cancer.

[123] Urbanczyk M, Layland SL, Schenke-Layland K (2020). The role of extracellular matrix in biomechanics and its impact on bioengineering of cells and 3D tissues. Matrix Biol.

[124] Dourado MR, Guerra ENS, Salo T, Lambert DW, Coletta RD (2018). Prognostic value of the immunohistochemical detection of cancer-associated fibroblasts in oral cancer: a systematic review and meta-analysis. J Oral Pathol Med.

[125] Lai SL, Tan ML, Hollows RJ, Robinson M, Ibrahim M, Margielewska S, Parkinson EK, Ramanathan A, Zain RB, Mehanna H et al. Collagen Induces a More Proliferative, Migratory and Chemoresistant Phenotype in Head and Neck Cancer via DDR1.Cancers (Basel)2019,11. 10.3390/cancers11111766 PMC6896141 31717573

[126] Hayashido Y, Kitano H, Sakaue T, Fujii T, Suematsu M, Sakurai S, Okamoto T (2014). Overexpression of integrin αv facilitates proliferation and invasion of oral squamous cell carcinoma cells via MEK/ERK signaling pathway that is activated by interaction of integrin αvβ8 with type I collagen. Int J Oncol.

[127] Hohenester E, Yurchenco PD (2013). Laminins in basement membrane assembly. Cell Adh Migr.

[128] Yellapurkar S, Natarajan S, Boaz K, Manaktala N, Baliga M, Shetty P, Prasad M, Ravi M (2018). Expression of laminin in oral squamous cell carcinomas. Asian Pac J Cancer Prev.

[129] Marangon Junior H, Rocha VN, Leite CF, de Aguiar MC, Souza PE, Horta MC (2014). Laminin-5 gamma 2 chain expression is associated with intensity of tumor budding and density of stromal myofibroblasts in oral squamous cell carcinoma. J Oral Pathol Med.

[130] Kinoshita T, Nohata N, Hanazawa T, Kikkawa N, Yamamoto N, Yoshino H, Itesako T, Enokida H, Nakagawa M, Okamoto Y, Seki N (2013). Tumour-suppressive microRNA-29s inhibit cancer cell migration and invasion by targeting laminin-integrin signalling in head and neck squamous cell carcinoma. Br J Cancer.

[131] Herbster S, Paladino A, de Freitas S, Boccardo E (2018). Alterations in the expression and activity of extracellular matrix components in HPV-associated infections and diseases. Clin (Sao Paulo).

[132] Yang SW, Tsai CY, Pan YC, Yeh CN, Pang JH, Takano M, Kittaka A, Juang HH, Chen TC, Chiang KC (2016). MART-10, a newly synthesized vitamin D analog, represses metastatic potential of head and neck squamous carcinoma cells. Drug Des Devel Ther.

[133] Vander Heiden MG, DeBerardinis RJ (2017). Understanding the Intersections between Metabolism and Cancer Biology. Cell.

[134] Lee N, Kim D (2016). Cancer Metabolism: fueling more than just growth. Mol Cells.

[135] Li Z, Zhang H (2016). Reprogramming of glucose, fatty acid and amino acid metabolism for cancer progression. Cell Mol Life Sci.

[136] Warburg O (1956). On the origin of cancer cells. Science.

[137] Ohashi T, Aoki M, Tomita H, Akazawa T, Sato K, Kuze B, Mizuta K, Hara A, Nagaoka H, Inoue N, Ito Y (2017). M2-like macrophage polarization in high lactic acid-producing head and neck cancer. Cancer Sci.

[138] Chang H, Xu Q, Li J, Li M, Zhang Z, Ma H, Yang X (2021). Lactate secreted by PKM2 upregulation promotes galectin-9-mediated immunosuppression via inhibiting NF-κB pathway in HNSCC. Cell Death Dis.

[139] Gates RE, Rees RS (1985). Altered vitamin A-binding proteins in carcinoma of the head and neck. Cancer.

[140] Won JY, Nam EC, Yoo SJ, Kwon HJ, Um SJ, Han HS, Kim SH, Byun Y, Kim SY (2004). The effect of cellular retinoic acid binding protein-I expression on the CYP26-mediated catabolism of all-trans retinoic acid and cell proliferation in head and neck squamous cell carcinoma. Metabolism.

[141] Lu H, Lu Y, Xie Y, Qiu S, Li X, Fan Z. Rational combination with PDK1 inhibition overcomes cetuximab resistance in head and neck squamous cell carcinoma.JCI Insight2019,4. 10.1172/jci.insight.131106 PMC6795401 31578313

[142] Petrova V, Annicchiarico-Petruzzelli M, Melino G, Amelio I (2018). The hypoxic tumour microenvironment. Oncogenesis.

[143] Asgari H, Soltani M, Sefidgar M (2018). Modeling of FMISO [F(18)] nanoparticle PET tracer in normal-cancerous tissue based on real clinical image. Microvasc Res.

[144] Brooks JM, Menezes AN, Ibrahim M, Archer L, Lal N, Bagnall CJ, von Zeidler SV, Valentine HR, Spruce RJ, Batis N (2019). Development and validation of a combined hypoxia and Immune Prognostic Classifier for Head and Neck Cancer. Clin Cancer Res.

[145] Nascimento-Filho CHV, Webber LP, Borgato GB, Goloni-Bertollo EM, Squarize CH, Castilho RM (2019). Hypoxic niches are endowed with a protumorigenic mechanism that supersedes the protective function of PTEN. Faseb j.

[146] Xu Q, Chang H, Tian X, Lou C, Ma H, Yang X (2020). Hypoxia-induced MFAP5 promotes Tumor Migration and Invasion via AKT Pathway in Head and Neck squamous cell carcinoma. J Cancer.

[147] Lin MC, Lin JJ, Hsu CL, Juan HF, Lou PJ, Huang MC (2017). GATA3 interacts with and stabilizes HIF-1α to enhance cancer cell invasiveness. Oncogene.

[148] Sun Q, Zhang SY, Zhao JF, Han XG, Wang HB, Sun ML (2020). HIF-1α or HOTTIP/CTCF promotes Head and Neck squamous cell Carcinoma Progression and Drug Resistance by Targeting HOXA9. Mol Ther Nucleic Acids.

[149] Wang R, Ma Z, Feng L, Yang Y, Tan C, Shi Q, Lian M, He S, Ma H, Fang J (2018). LncRNA MIR31HG targets HIF1A and P21 to facilitate head and neck cancer cell proliferation and tumorigenesis by promoting cell-cycle progression. Mol Cancer.

[150] Gong L, Zhang W, Zhou J, Lu J, Xiong H, Shi X, Chen J (2013). Prognostic value of HIFs expression in head and neck cancer: a systematic review. PLoS ONE.

[151] Sacco AG, Chen R, Worden FP, Wong DJL, Adkins D, Swiecicki P, Chai-Ho W, Oppelt P, Ghosh D, Bykowski J (2021). Pembrolizumab plus cetuximab in patients with recurrent or metastatic head and neck squamous cell carcinoma: an open-label, multi-arm, non-randomised, multicentre, phase 2 trial. Lancet Oncol.

[152] Gillison ML, Trotti AM, Harris J, Eisbruch A, Harari PM, Adelstein DJ, Jordan RCK, Zhao W, Sturgis EM, Burtness B (2019). Radiotherapy plus cetuximab or cisplatin in human papillomavirus-positive oropharyngeal cancer (NRG Oncology RTOG 1016): a randomised, multicentre, non-inferiority trial. Lancet.

[153] Guigay J, Aupérin A, Fayette J, Saada-Bouzid E, Lafond C, Taberna M, Geoffrois L, Martin L, Capitain O, Cupissol D (2021). Cetuximab, docetaxel, and cisplatin versus platinum, fluorouracil, and cetuximab as first-line treatment in patients with recurrent or metastatic head and neck squamous-cell carcinoma (GORTEC 2014-01 TPExtreme): a multicentre, open-label, randomised, phase 2 trial. Lancet Oncol.

[154] Burtness B, Rischin D, Greil R, Soulières D, Tahara M, de Castro G, Psyrri A, Brana I, Basté N, Neupane P (2022). Pembrolizumab alone or with chemotherapy for Recurrent/Metastatic Head and Neck squamous cell carcinoma in KEYNOTE-048: subgroup analysis by programmed death Ligand-1 combined positive score. J Clin Oncol.

[155] Muraro E, Fanetti G, Lupato V, Giacomarra V, Steffan A, Gobitti C, Vaccher E, Franchin G (2021). Cetuximab in locally advanced head and neck squamous cell carcinoma: Biological mechanisms involved in efficacy, toxicity and resistance. Crit Rev Oncol Hematol.

[156] Alsahafi E, Begg K, Amelio I, Raulf N, Lucarelli P, Sauter T, Tavassoli M (2019). Clinical update on head and neck cancer: molecular biology and ongoing challenges. Cell Death Dis.

[157] Chen X, Song E. The theory of tumor ecosystem.Cancer Commun (Lond)2022. 10.1002/cac2.12316 PMC9257988 35642770

[158] Pauli C, Hopkins BD, Prandi D, Shaw R, Fedrizzi T, Sboner A, Sailer V, Augello M, Puca L, Rosati R (2017). Personalized in Vitro and in vivo Cancer models to Guide Precision Medicine. Cancer Discov.

[159] Yao Y, Wang Y, Chen L, Tian Z, Yang G, Wang R, Wang C, Wu Q, Wu Y, Gao J (2022). Clinical utility of PDX cohorts to reveal biomarkers of intrinsic resistance and clonal architecture changes underlying acquired resistance to cetuximab in HNSCC. Signal Transduct Target Ther.

[160] Li Y, Goldberg EM, Chen X, Xu X, McGuire JT, Leuzzi G, Karagiannis D, Tate T, Farhangdoost N, Horth C (2022). Histone methylation antagonism drives tumor immune evasion in squamous cell carcinomas. Mol Cell.

[161] Fazio M, Ablain J, Chuan Y, Langenau DM, Zon LI (2020). Zebrafish patient avatars in cancer biology and precision cancer therapy. Nat Rev Cancer.

[162] Hagege A, Ambrosetti D, Boyer J, Bozec A, Doyen J, Chamorey E, He X, Bourget I, Rousset J, Saada E (2021). The Polo-like kinase 1 inhibitor onvansertib represents a relevant treatment for head and neck squamous cell carcinoma resistant to cisplatin and radiotherapy. Theranostics.

[163] Hagege A, Saada-Bouzid E, Ambrosetti D, Rastoin O, Boyer J, He X, Rousset J, Montemagno C, Doyen J, Pedeutour F (2022). Targeting of c-MET and AXL by cabozantinib is a potential therapeutic strategy for patients with head and neck cell carcinoma. Cell Rep Med.

[164] Kartha VK, Alamoud KA, Sadykov K, Nguyen BC, Laroche F, Feng H, Lee J, Pai SI, Varelas X, Egloff AM (2018). Functional and genomic analyses reveal therapeutic potential of targeting β-catenin/CBP activity in head and neck cancer. Genome Med.

[165] Sachs N, Clevers H (2014). Organoid cultures for the analysis of cancer phenotypes. Curr Opin Genet Dev.

[166] Driehuis E, Kolders S, Spelier S, Lõhmussaar K, Willems SM, Devriese LA, de Bree R, de Ruiter EJ, Korving J, Begthel H (2019). Oral mucosal organoids as a potential platform for Personalized Cancer Therapy. Cancer Discov.

[167] Qi Z, Barrett T, Parikh AS, Tirosh I, Puram SV (2019). Single-cell sequencing and its applications in head and neck cancer. Oral Oncol.

[168] Blise KE, Sivagnanam S, Banik GL, Coussens LM, Goecks J (2022). Single-cell spatial architectures associated with clinical outcome in head and neck squamous cell carcinoma. NPJ Precis Oncol.

[169] Sun L, Kang X, Wang C, Wang R, Yang G, Jiang W, Wu Q, Wang Y, Wu Y, Gao J (2023). Single-cell and spatial dissection of precancerous lesions underlying the initiation process of oral squamous cell carcinoma. Cell Discov.

[170] Kann BH, Hicks DF, Payabvash S, Mahajan A, Du J, Gupta V, Park HS, Yu JB, Yarbrough WG, Burtness BA (2020). Multi-institutional validation of deep learning for pretreatment identification of Extranodal Extension in Head and Neck squamous cell carcinoma. J Clin Oncol.

[171] Puladi B, Ooms M, Kintsler S, Houschyar KS, Steib F, Modabber A, Hölzle F, Knüchel-Clarke R, Braunschweig T. Automated PD-L1 Scoring Using Artificial Intelligence in Head and Neck Squamous Cell Carcinoma.Cancers (Basel)2021,13. 10.3390/cancers13174409 PMC8431396 34503218

[172] Klein S, Quaas A, Quantius J, Löser H, Meinel J, Peifer M, Wagner S, Gattenlöhner S, Wittekindt C, von Knebel Doeberitz M (2021). Deep learning predicts HPV Association in Oropharyngeal squamous cell carcinomas and identifies patients with a favorable prognosis using regular H&E stains. Clin Cancer Res.

[173] Singh AK, Ling J, Malviya SAU (2023). Prediction of Cancer Treatment Using Advancements in Machine Learning. Recent Pat Anticancer Drug Discov.

[174] Azam MA, Sampieri C, Ioppi A, Benzi P, Giordano GG, De Vecchi M, Campagnari V, Li S, Guastini L, Paderno A (2022). Videomics of the Upper Aero-Digestive Tract Cancer: Deep Learning Applied to White Light and narrow Band Imaging for Automatic segmentation of endoscopic images. Front Oncol.

[175] Wei LY, Lee JJ, Yeh CY, Yang CJ, Kok SH, Ko JY, Tsai FC, Chia JS (2019). Reciprocal activation of cancer-associated fibroblasts and oral squamous carcinoma cells through CXCL1. Oral Oncol.

[176] Li X, Bu W, Meng L, Liu X, Wang S, Jiang L, Ren M, Fan Y, Sun H (2019). CXCL12/CXCR4 pathway orchestrates CSC-like properties by CAF recruited tumor associated macrophage in OSCC. Exp Cell Res.

[177] Ansel KM, Harris RB, Cyster JG (2002). CXCL13 is required for B1 cell homing, natural antibody production, and body cavity immunity. Immunity.

[178] Jiffar T, Yilmaz T, Lee J, Miller Y, Feng L, El-Naggar A, Kupferman ME. Brain derived neutrophic factor (BDNF) coordinates lympho-vascular metastasis through a fibroblast-governed paracrine axis in the tumor microenvironment.Cancer Cell Microenviron2017,4. 10.14800/ccm.1566 PMC5617346 28966935

[179] Wang Y, Jing Y, Ding L, Zhang X, Song Y, Chen S, Zhao X, Huang X, Pu Y, Wang Z (2019). Epiregulin reprograms cancer-associated fibroblasts and facilitates oral squamous cell carcinoma invasion via JAK2-STAT3 pathway. J Exp Clin Cancer Res.

[180] Knowles LM, Stabile LP, Egloff AM, Rothstein ME, Thomas SM, Gubish CT, Lerner EC, Seethala RR, Suzuki S, Quesnelle KM (2009). HGF and c-Met participate in paracrine tumorigenic pathways in head and neck squamous cell cancer. Clin Cancer Res.

[181] Haga K, Yamazaki M, Maruyama S, Kawaharada M, Suzuki A, Hoshikawa E, Chan NN, Funayama A, Mikami T, Kobayashi T (2021). Crosstalk between oral squamous cell carcinoma cells and cancer-associated fibroblasts via the TGF-β/SOX9 axis in cancer progression. Transl Oncol.

[182] Zhang Z, Neiva KG, Lingen MW, Ellis LM, Nör JE (2010). VEGF-dependent tumor angiogenesis requires inverse and reciprocal regulation of VEGFR1 and VEGFR2. Cell Death Differ.

